# Gallnut Tannic Acid Exerts Anti-stress Effects on Stress-Induced Inflammatory Response, Dysbiotic Gut Microbiota, and Alterations of Serum Metabolic Profile in Beagle Dogs

**DOI:** 10.3389/fnut.2022.847966

**Published:** 2022-04-27

**Authors:** Kang Yang, Shiyan Jian, Chaoyu Wen, Dan Guo, Pinfeng Liao, Jiawei Wen, Tao Kuang, Sufang Han, Qingshen Liu, Baichuan Deng

**Affiliations:** Guangdong Laboratory for Lingnan Modern Agriculture, Guangdong Provincial Key Laboratory of Animal Nutrition Control, National Engineering Research Center for Breeding Swine Industry, College of Animal Science, South China Agricultural University, Guangzhou, China

**Keywords:** gallnut tannic acid, stress exposure, inflammatory response, gut microbiota, serum metabolomics, beagle dog

## Abstract

Stress exposure is a potential threat to humans who live or work in extreme environments, often leading to oxidative stress, inflammatory response, intestinal dysbiosis, and metabolic disorders. Gallnut tannic acid (TA), a naturally occurring polyphenolic compound, has become a compelling source due to its favorable anti-diarrheal, anti-oxidative, anti-inflammatory, and anti-microbial activities. Thus, this study aimed to evaluate the anti-stress effects of gallnut TA on the stress-induced inflammatory response, dysbiotic gut microbiota, and alterations of serum metabolic profile using beagle models. A total of 13 beagle dogs were randomly divided into the stress (ST) and ST + TA groups. Dietary supplementation with TA at 2.5 g/kg was individually fed to each dog in the ST + TA group for 14 consecutive days. On day 7, all dogs were transported for 3 h from a stressful environment (days 1–7) to a livable site (days 8–14). In our results, TA relieved environmental stress-induced diarrheal symptoms in dogs and were shown to protect from myocardial injury and help improve immunity by serum biochemistry and hematology analysis. Also, TA inhibited the secretion of serum hormones [cortisol (COR), glucocorticoid (GC), and adrenocorticotropic hormone (ACTH)] and the expression of heat shock protein (HSP) 70 to protect dogs from stress-induced injury, thereby relieving oxidative stress and inflammatory response. Fecal 16S rRNA gene sequencing revealed that TA stimulated the growth of beneficial bacteria (*Allobaculum*, *Dubosiella*, *Coriobacteriaceae_UCG-002*, and *Faecalibaculum*) and suppressed the growth of pathogenic bacteria (*Escherichia-Shigella* and *Streptococcus*), thereby increasing fecal butyrate levels. Serum metabolomics further showed that phytosphingosine, indoleacetic acid, arachidonic acid, and biotin, related to the metabolism of sphingolipid, tryptophan, arachidonic acid, and biotin, respectively, could serve as potential biomarkers of stress exposure. Furthermore, Spearman’s correlation analysis showed strong relationships between the four potential serum biomarkers and differential bacteria. Overall, gallnut TA may be a potential prebiotic for the prevention and treatment of stress-induced metabolic disorders by targeting intestinal microbiota.

## Introduction

Stress is the normal physiological response of the body to environmental or psychological changes (1). Substances that induce stresses, such as heat, chemical, and physiological stress, are called stressors (2, 3). Stress exposure is a potential threat to humans who are living or working in extreme environments, and numerous studies have reported that lots of stresses have negative influences on the organisms and induce physiological and pathophysiological damages, including oxidative stress, inflammation, and neuronal apoptosis (4–6). During stressful events, the secretion of stress hormones was elevated (7, 8). Numerous studies have confirmed that the hypothalamic-pituitary-adrenal (HPA) axis response is activated by stress, thereby releasing stress hormones, such as cortisol (COR) (9–11). Additionally, heat shock proteins (HSPs), well-conserved molecules of transcriptional regulators, could respond to various stressors to prevent apoptotic processes in different cell types (12, 13). As the relationship among the gut, the brain, and gut microbiota is interconnected, stress-triggered activation of the sympathetic nervous system and the HPA axis affects the microbiota that inhabits the gastrointestinal tract (14).

Gut dysbiosis, caused by stress, leads to impaired health, including disruption of the intestinal barrier and enhanced pro-inflammatory response (15). Stress-induced overgrowth of pathogenic bacteria results in the disturbance of intestinal microbiota and the disruption of immune system, causing intestinal inflammation, depression, and cognitive impairment through the induction of IL-1β and corticosterone secretion (16, 17). Indeed, recent studies have revealed that the shifts in intestinal microbiota composition might be the reason for stress-induced gastrointestinal symptoms (18, 19). In addition, intestinal microecology not only contains gut microbiota but also a large number of gut microbiota metabolites, such as short-chain fatty acids (SCFAs). The reduction of colonic pH by SCFAs was found to promote colonic health (20, 21). One study in mice showed that stress exposure reduced colonic SCFAs levels and changed the relative abundances of SCFAs-producing bacteria (22). Therefore, increasing SCFAs levels could alleviate stress-induced brain-gut axis alterations (23). In brief, gut microbiota and its metabolites influence host immune function, and normal gut microbiota is important for the maintenance of intestinal homeostasis (24).

Recent studies suggest that polyphenols may be promising candidates for prebiotics (25, 26). There is strong evidence that dietary polyphenol compounds can stimulate the growth of beneficial intestinal bacteria, such as *Lactobacillus*, *Bifidobacterium*, *Akkermansia*, *Roseburia*, and *Faecalibacterium* spp. (27), and the production of SCFAs. Thus, polyphenols exert prebiotic actions and inhibit the growth of pathogenic bacteria (26, 28, 29). As a widely used traditional medicine in China, gallnut (*Galla chinensis*) is rich in tannic acid (TA), even accounting for 50–70% of its weight (30). Gallnut TA belongs to the family of hydrolyzable tannins (31) and is a naturally occurring polyphenol compound of high molecular weight (500–3,000 Da). The structures of gallnut TA are made up of a polyol core (typically D-glucose), which is esterified with phenolic acids (mainly gallic acid or hexahydroxy diphenic acid) (32). The TA is generally considered an anti-nutritional factor (33, 34). Interestingly, because of its polyphenolic hydroxyl structure, TA has various biological activities, including anti-diarrheal, anti-oxidative, anti-microbial, anti-parasitic, and anti-cancer (35, 36). Some studies reported that supplementing TA with appropriate amounts had a beneficial effect on relieving diarrhea without negative impacts on the growth performance (37, 38). Thus, TA has great potential to balance and normalize gut microbiota to keep the host healthy. However, knowledge of the influence of diet supplemented with TA on stress is still limited.

In this study, beagle dogs were selected because of the high similarity of the gut microbiome of dogs and humans in terms of genetic content and response to diet (39). Our previous study explored the effect of changing environment and addition of gallic acid on stressful puppies (40). Next, this study aims to compare the effect of TA on stressful puppies to confirm whether TA works by hydrolysis to gallic acid. In addition, considering the correlation between stress and gut microbiota alterations, we hypothesized that TA could relieve stress by modulating intestinal microbiota disorder and host metabolism. Therefore, microbiome and metabolomics were adopted to reveal a relationship between gut microbiota and its metabolites in dogs supplemented with TA. Overall, the present study was conducted to investigate whether TA influences the diarrhea rate, inflammatory response, fecal microbiota, and serum metabolic profiles in dogs.

## Materials and Methods

### Materials

**FIGURE 1:**
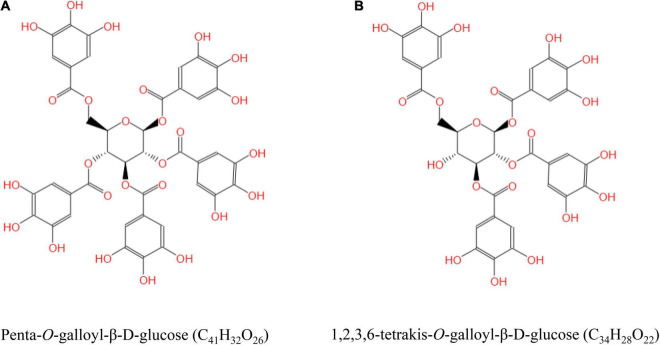
The structural formula of tannic acid (TA). Penta-*O*-galloyl-β-D-glucose (C_41_H_32_O_26_) **(A)** and 1,2,3,6-tetrakis-*O*-galloyl-β-D-glucose (C_34_H_28_O_22_) **(B)**.

Tannic acid (purity > 93%) extracted from gallnut was purchased from Wufeng Chicheng Biotech Co., Ltd. (Yichang, China). The gallnut samples identified by UPLC-Orbitrap-MS/MS confirmed that the chemical formulas of TA are C_41_H_32_O_26_ and C_34_H_28_O_22_, and its structural formulas are shown in Figure 1. Beagle dogs with average weight of 5.23 ± 0.62 kg and average age of 3.56 ± 0.24 months were bought from the National Canine Laboratory Animal Resource Bank, Guangzhou General Pharmaceutical Research Institute Co., Ltd. (Guangzhou, China), license number: SCXK (Guangdong) 2018-0007. Basal diet (commercially extruded feed, dry matter: 90.5%, organic matter: 92.8%, protein: 23.9%, fat: 4.6%, fiber: 3.9%, and gross energy: 17.0 kJ/g) was manufactured at the Dongguan Yinhua Bio-Tech Co., Ltd. (Dongguan, China), license number: SCXK (Guangdong) 2019-11016. Commercial kits for measuring the levels of glutathione (GSH), peroxidase (GSH-Px), malondialdehyde (MDA), total anti-oxidant capacity (T-AOC), and superoxide dismutase (SOD) were obtained from Nanjing Jiancheng Bioengineering Institute (Nanjing, China). Serum COR (product no. MM-32604O1), glucocorticoid (GC, MM-2277O1), adrenocorticotropic hormone (ACTH, MM-1739O1), HSP-70 (MM-85074O1), immunoglobulin G (IgG, MM-2086O1), tumor necrosis factor-α (TNF-α, MM-36988O1), interferon-γ (IFN-γ, MM-35063O1), and interleukin-4 (IL-4, MM-35084O1) were measured using commercial canine enzyme-linked immunosorbent assay (ELISA) kits (MEIMIAN, Jiangsu Meimian Industrial Co., Ltd., Jiangsu, China).

### Animals and Experimental Design

The study was approved by the Experimental Animal Ethics Committee of South China Agricultural University (protocol code 2021E028). A total of 13 beagle dogs were selected in this study and were caged individually in a room maintained at 29°C ± 1°C with a relative humidity of 96% ± 3%, and a 12-h light/dark cycle at Guangzhou General Pharmaceutical Research Institute Co., Ltd. (Guangzhou, China). Water was freely available. Taking into account the dog’s habit of overeating, the puppies were fed a restricted amount of commercial extruded feed (basal diet), in equal amounts (100 g), at 08:00 and 17:00. The basal diet meets or exceeds the nutrient recommendations by the Association of American Feed Control Officials (AAFCO, 2017) for puppies (41). After a month of adaptation, the dogs were randomly divided into two groups: stress group (ST, *n* = 6) and ST + TA group (*n* = 7). The experiment period lasted for 2 weeks. Gallnut TA was mixed with the basal diet and individually fed to each dog in the ST + TA group at 2.5 g/kg (equivalent to 0.5 g per 200 g) for 14 consecutive days. The dosage of TA supplements was slightly modified based on previous studies (42, 43). Feed consumption per dog was consistently monitored to ensure the feed intake. On day 7, all dogs were transported for 3 h to the Laboratory Animal Center Building at the South China Agricultural University and were housed individually in pens (1.2 m × 1.0 m × 1.1 m kennels) under constant temperature and humidity (23^°^C, 70%) with a 12-h light/dark cycle. Both groups continued on their diets for another week. Fecal score (FS) (44) were adopted to assess daily fecal form and consistency. The weight and body condition score (BCS) assessed using a nine-point scale (45) were recorded separately on days 1, 4, and 7 (before transportation) and days 8, 11, and 14 (after transportation) in the morning before feeding.

### Blood Routine Examination and Serum Biochemical Analyses

On days 1, 7, 8, and 14 after overnight fasting, blood samples were harvested and blood routine examination was performed using a Mindray^®^ automatic hematology analyzer (BC-2800vet, Shenzhen Mindray Bio-Medical Electronics Co., Ltd., Shenzhen, China). The serum biochemical parameters were detected by an automatic blood biochemical analyzer (Chemray 800, Shenzhen Redu Life Technology, Shenzhen, China). Serum GSH, GSH-Px, MDA, T-AOC, and SOD were detected using commercial kits according to the manufacturer’s protocol. Serum COR, GC, ACTH, HSP-70, IgG, TNF-α, IFN-γ, and IL-4 were measured using commercial canine ELISA kits.

### Fecal Microbiota Analysis

On days 7, 8, and 14, fresh fecal samples were collected from the metabolic cages of each dog within 15 min of defecation and transferred to a 5-ml sterile fecal collection tube, then snap-frozen on liquid N_2_ and stored at -80^°^C until DNA extraction. Microbial genomic DNA from fecal samples was extracted using the CTAB/SDS method. After monitoring the DNA concentration and purity on 1% agarose gels, DNA was diluted to 1 ng/μl with sterile water. The V3-V4 region of the bacterial 16S rRNA gene was amplified using the specific primers 341F (5′-CCTAYGGGRBGCASCAG-3′) and 806R (5′-GGACTACNNGGGTATCTAAT-3′) with the barcode. All PCR reactions were carried out with 15 μl of Phusion^®^ High-Fidelity PCR Master Mix (New England Biolabs) with 0.2 μM of forward and reverse primers and 10-ng template DNA, and cycling conditions consisted of a first denaturation step at 98^°^C for 1 min, followed by 30 cycles at 98^°^C (10 s), 50^°^C (30 s), and 72^°^C (30 s) and a final 5-min extension at 72^°^C. An equal volume of 1X loading buffer (contained SYB green) was mixed with PCR products, and electrophoresis was performed on 2% agarose gel for DNA detection. The PCR products were mixed in equal proportions, and then the mixed PCR products were purified using the Qiagen Gel Extraction kit (Qiagen, Germany). Sequencing libraries were generated using the TruSeq^®^ DNA PCR-Free Sample Preparation kit (Illumina, United States). The library quality was assessed on the Qubit@ 2.0 Fluorometer (Thermo Scientific) and Agilent Bioanalyzer 2100 system. Finally, the library was sequenced on an Illumina NovaSeq platform and 250-bp paired-end reads were generated. Paired-end reads were assigned to samples based on their unique barcode and truncated by cutting off the barcode and primer sequence, and paired-end reads were merged using FLASH (version 1.2.11) (46). Next, quality filtering on the raw tags was performed to obtain the high-quality clean tags according to the fastp (version 0.20.0) software. Then, the clean tags were compared with the Silva database for 16S using Vsearch (version 2.15.0), and the chimera sequences were removed to obtain the effective tags (47).

For the effective tags, denoise was performed with DADA2 in the QIIME2 software (version QIIME2-202006) to obtain initial amplicon sequence variants (ASVs), and then ASVs with abundance less than 5 were filtered out (48). Species annotation was performed using the QIIME2 software. To study the phylogenetic relationship of each ASV and the differences of the dominant species among different samples (groups), a multi-sequence alignment was performed using the QIIME2 software. The absolute abundance of ASVs was normalized using a standard sequence number corresponding to the sample with the least sequences. The Venn analysis was performed by the Venn diagram website.^1^ All subsequent analyses of α- and β-diversity were performed based on the output normalized data. α-diversity (Observed_species, Chao1, Shannon, Simpson, Pielou_e, Dominance, and Good’s coverage) and β-diversity were calculated in QIIME2. The two-dimensional principal coordinate analysis (PCoA) based on the weighted unifrac distances results were displayed using the ade4 package and ggplot2 package in R software (version 2.15.3).

The linear discriminant analysis effect size (LEfSe) software (version 1.0) was used to perform an LEfSe analysis [linear discriminant analysis (LDA) score > 3] to find out the biomarkers. A clustered heatmap with the rank abundance plot of bacterial genera was plotted using the R software (version 3.1.0). The relationship between microbial community composition and environmental factors was explained through the redundancy analysis (RDA) of the vegan package (2.5–7) using R software (version 3.6.3), and visualization was performed by the OmicStudio tools at https://www.omicstudio.cn/tool.

### Fecal Short-Chain Fatty Acids and Branched-Chain Fatty Acids Analyses

Short-chain fatty acids and branched-chain fatty acids (BCFAs) concentrations in fecal samples on days 7, 8, and 14 were determined using the GCMS-QP2020 system (Shimadzu, Tokyo, Japan) with a DB-FFAP capillary column (30 m × 0.25 mm × 0.25 μm, Onlysci, China). Instrument parameters and fecal sample pre-processing methods referred to our previous study (40). Briefly, the program was run as follows: the initial temperature of the column was 80^°^C for 2 min, increased to 150^°^C at a rate of 10^°^C/min for 2 min, and increased to 180^°^C at a rate of 15^°^C/min for 5 min. The total run time was 18 min. The injection port temperature was set at 250^°^C. The sample injection volume was 0.6 μl with a 30:1 split ratio. Helium (He, 99.999%) was the carrier gas with a flow rate of 3 ml/min. The MS parameters were electron impact modes with ionization energy of 70 eV. Ion source and interface temperatures were 230 and 250^°^C, respectively. Sample preparation was done as follows: the frozen stool samples were placed on ice to thaw, and 0.2 g of feces was added with 1 ml of ultrapure water. After vortex for 2 min, the samples were sonicated in an ice bath for 10 min, then centrifuged at 14,000 rpm for 10 min at 4^°^C. The supernatant was immediately transferred to a 2-ml centrifuge tube, and then a total of 20-μl 25% metaphosphoric acid solution and 0.25 g of anhydrous sodium sulfate were added for acidification and salting out, respectively. After vortex for 2 min, 1 ml of methyl tert-butyl ether was added, then vortex was continued for 5 min, and the supernatant was further centrifuged at 14,000 rpm for another 10 min at 4^°^C. Finally, the supernatant was harvested and filtered through 0.22-μm Millipore membrane filters to a 2-ml sample bottle.

### Serum Metabolomics Analysis

Frozen serum samples collected on days 7, 8, and 14 were thawed at 4°C, and vortex-mixed for 2 min. Then, 200 ml of the serum sample and 800 ml of methanol were sequentially added to a 1.5-ml sterile DNAase- and RNAase-free Eppendorf tube, and vortex-mixed for 2 min. Then, the samples were centrifuged at 14,500 rpm, 4°C for 15 min, and an 800-ml supernatant was blow-dried with nitrogen. Around 100 ml of supernatant from each sample was mixed to obtain a quality control sample. Next, the samples were redissolved with 200 ml of methanol and vortex-mixed for 2 min. Ultrasonic crushing was performed at a low temperature for 10 min, and then all samples were centrifuged at 14,500 rpm, 4°C for 15 min. Finally, the samples were filtered through 0.22-mm microporous membranes for the UPLC-Orbitrap-MS/MS analysis.

The UPLC-Orbitrap-MS/MS analysis method was conducted as described previously (50) with slight modifications. The Compound Discoverer 2.1 (Thermo Fisher Scientific) data analysis tool was employed to automatically complete raw data pre-processing and was applied to identify metabolites by searching the mzCloud library and mzVault library. In this study, MetaboAnalyst 5.0^2^ was used to perform a multivariate analysis. The principal component analysis (PCA) of metabolites was performed. A pathway enrichment analysis was performed by using the enrichment analysis module on MetaboAnalyst 5.0. The visualization results of the models were obtained with MetaboAnalyst 5.0.

### Statistical Analysis

SPSS 26.0 and GraphPad Prism 8.0 software were used for statistical analysis and graphical display. A comparison between the two groups was performed by Student’s *t*-test. For repeated-measure testing, repeated-measure analysis of variance (RM-ANOVA) with Bonferroni adjustment for multiple comparisons was performed to analyze the differences within each group at varying time points. All data were expressed as the mean ± standard error (SE). Significant differences were obtained at *p* < 0.05, and tendencies were obtained at *p* < 0.10. To preliminarily screen differential metabolites, we selected the metabolites that had a *p* < 0.05 and fold change (FC) values > 2 or < 0.5. The two-way orthogonal partial least squares (O2PLS) method consists of the simultaneous projection of both X and Y matrices on low-dimension hyper planes (51). To reveal the association between microbiome and metabolomics, bacterial genera with relative abundance > 0.1% (X matrix) and 147 serum metabolites (Y matrix) were observed using the O2PLS analysis, in which the X matrix was mapped to Y matrix. The correlation matrix shows a pair-wise correlation among all variables (X and Y), in which the value of correlation coefficient represents the extent of the linear association between the two terms, ranging from -1 to 1. The O2PLS analysis and graphics were performed using the OmicShare tools at https://www.omicshare.com/tools/Home/Soft/o2pls. Spearman’s correlation values and significance were computed with R version 3.6.1. Clustering correlation heatmap with signs and advanced volcano plot were generated using the OmicStudio tools at https://www.omicstudio.cn/tool.

## Results

### Effect of Tannic Acid on Body Condition, Fecal Score, Hematology, and Serum Biochemistry

**FIGURE 2:**
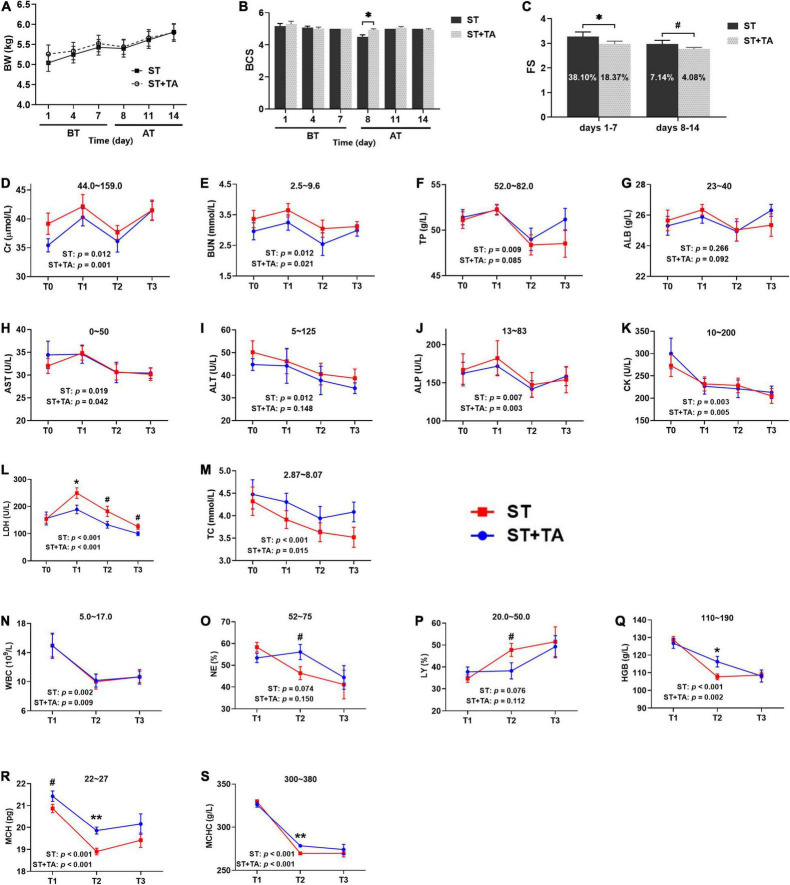
Effect of TA on body condition **(A,B)**, fecal score (FS) **(C)**, serum biochemistry **(D–M)**, and hematology **(N–S)** in dogs. Data are presented as mean ± standard error (SE) (*n* = 6 or 7). *p < 0.05 and ^**^p < 0.01 represent the difference calculated by Student’s *t*-test between the stress (ST) and ST + TA groups; ^#^*p* < 0.10 represents the difference in tendency calculated by Student’s *t*-test between the ST and ST + TA groups. The *p*-values in the figures indicate the difference of each group at T1–T3 calculated by repeated-measure analysis of variance (RM-ANOVA). Numbers in the column chart indicate the diarrhea rate. Numbers at the top of each figure refer to the normal reference ranges for serum biochemistry and hematology indices. BT, before transportation; AT, after transportation; T0, day 1 before transportation; T1, day 7 before transportation; T2, day 8 after transportation; T3, day 14 after transportation. Cr, creatinine; BUN, blood urea nitrogen; TP, total protein; ALB, albumin; AST, aspartate aminotransferase; ALT, alanine aminotransferase; ALP, alkaline phosphatase; CK, creatine kinase; LDH, lactate dehydrogenase; TC, total cholesterol; WBC, white blood cell count; NE, neutrophils; LY, lymphocyte; HGB, hemoglobin; MCH, mean corpuscular hemoglobin; MCHC, mean corpuscular hemoglobin concentration.

Body weight, BCS, and FS reflect the general health state of dogs. No difference was observed in body weight (Figure 2A), but BCS in the ST group showed an evident decrease (*p* < 0.05) than that in the ST + TA group on day 8 (T2) (Figure 2B), indicating that the supplementation of TA could maintain the dogs’ good body condition. The result of FS was represented in Figure 2C, showing that dogs fed TA markedly reduced (*p* < 0.05) the FS on days 1–7 before transportation and had a decreasing trend (*p* < 0.1) on days 8–14 after transportation. Furthermore, the diarrhea rate in dogs dropped from 38.10 to 18.37% (days 1–7) and 7.14 to 4.08% (days 8–14). This suggested that dogs transferred to a livable environment had a normal fecal shape and lower diarrhea rate compared with the original stressful environment, and dogs fed TA at 2.5 g/kg further reduced the diarrhea rate.

Serum biochemistry reflective of renal function [creatinine (Cr) and blood urea nitrogen (BUN)], liver function [total protein (TP) and albumin (ALB)], liver injury [aspartate transaminase (AST), alanine transaminase (ALT), and alkaline phosphatase (ALP)], myocardial injury [creatine kinase (CK) and lactate dehydrogenase (LDH)], and lipid metabolism (TC) were assessed in dogs (Figures 2D–M). Higher (*p* < 0.05) serum Cr, BUN, TP, AST, ALT, ALP, CK, LDH, and TC levels of each group were observed at T0 or T1 before transportation than those at T2 or T3 after transportation. In addition, dietary supplementation of TA had lower (*p* < 0.1) LDH levels compared with the ST group at T1–T3. Collectively, a stressful environment would cause liver, kidney, and myocardial injury in dogs. These symptoms could be alleviated when transported to a new livable site, and TA had a further protective effect on myocardial injury in dogs.

High white blood cells [WBC, including neutrophils (NE) and lymphocytes (LY)] is a reflection of inflammation, and hemoglobin [HGB, including mean corpuscular hemoglobin (MCH) and mean corpuscular hemoglobin concentration (MCHC)] content indirectly reflects the immunity level (Figures 2N–S). In each group, the levels of WBC, NE, HGB, MCH, and MCHC were higher (*p* < 0.05) at T1 than at T2 or T3. The percentage of NE and LY in the ST group almost exceeded its normal range after transportation; however, these values returned to normal after supplementation with TA (*p* < 0.1). It was obvious that HGB, MCH, and MCHC concentrations were below the normal range after transportation, while they significantly increased (*p* < 0.05) in dogs fed TA. Viewed as a whole, stressful environment contributed to waning immunity in dogs, whereas both changing living environment and TA supplementation help to enhance immunity.

### Effect of Tannic Acid on Heat Stress Protein-70, Hormone, Inflammation, and Anti-oxidant

**FIGURE 3:**
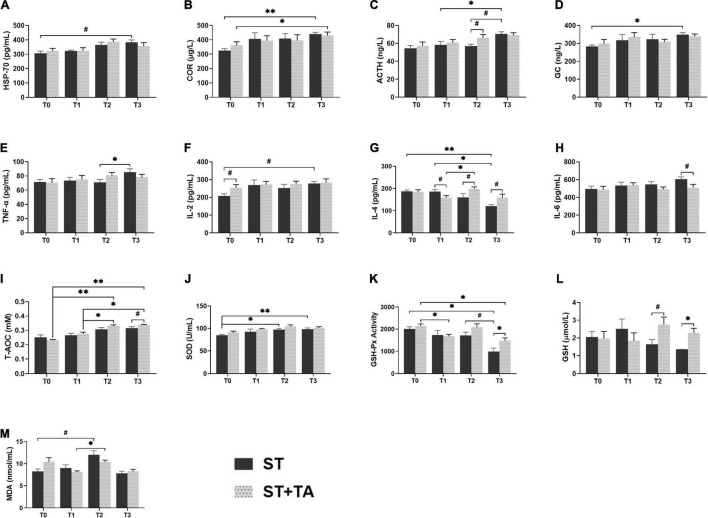
Effect of TA on HSP-70 **(A)**, hormone **(B–D)**, inflammation **(E–H)**, and anti-oxidant **(I–M)** in dogs. Data are presented as mean ± SE (*n* = 6 or 7). *p < 0.05 and ^**^p < 0.01 represent the difference calculated by Student’s *t*-test between the ST and ST + TA groups or the difference of each group at T0–T3 calculated by RM-ANOVA; ^#^*p* < 0.10 represents the difference in tendency calculated by Student’s *t*-test between the ST and ST + TA groups or the difference in tendency of each group at T0–T3 calculated by RM-ANOVA. T0, day 1 before transportation; T1, day 7 before transportation; T2, day 8 after transportation; T3, day 14 after transportation; HSP-70, heat stress protein 70; COR, cortisol; ACTH, adreno-cortico-tropic-hormone; GC, glucocorticoid; TNF-α, tumor necrosis factor-α; IL-2, interleukin-2; IL-4, interleukin-4; IL-6, interleukin-6; T-AOC, total anti-oxidant capacity; SOD, superoxide dismutase; GSH-Px, glutathione peroxidase; GSH, glutathione; MDA, malondialdehyde.

Heat stress protein-70 (HSP-70), a kind of stress-induced protein, protects cells against stresses, and serum COR, ACTH, and GC were considered as the stress hormones in response to various environmental stressors. In the ST group, dogs had an increasing trend (*p* < 0.1) in HSP-70 at T3 compared with T0, while this trend was not observed in the ST + TA group (Figure 3A). The highest levels of serum COR (*p* < 0.01), ACTH (*p* < 0.05), and GC (*p* < 0.05) were observed at T3 in the ST group, while supplementation with TA showed an evident increase (*p* < 0.05) of COR at T3 compared with T0, and had an increasing trend (*p* < 0.1) toward ACTH at T2 compared with the ST group (Figures 3B–D). The results indicated that TA might have the potential to alleviate stress in dogs after transportation.

Inflammation and anti-oxidant indicators can reflect the body’s inflammatory state and redox level. In the ST group, we observed the elevated (*p* < 0.05) TNF-α at T3 compared with T2, and an increasing trend (*p* < 0.1) in IL-2 level at T3 compared with T0 (Figures 3E,F). Compared with pre-transportation, the IL-4 content was significantly decreased (*p* < 0.05) in the ST group, while dogs fed TA had an increasing (*p* < 0.1) trend after transportation (Figure 3G). There was a decreasing trend (*p* < 0.1) in IL-6 content at T3 in the ST + TA group relative to the ST group (Figure 3H). Additionally, we also found T-AOC levels at T2 and T3 were significantly higher (*p* < 0.05) than that at T0 and T1 in the ST + TA group, and dogs fed TA had an increasing trend (*p* < 0.1) of T-AOC at T3 compared with the ST group (Figure 3I). The ST group had a higher (*p* < 0.05) SOD level when dogs were transported to a livable site (Figure 3J). Though lower (*p* < 0.05) GSH-Px activity of each group was observed after transportation, the supplementation of TA had a higher (*p* < 0.05) GSH-Px activity than that in the ST group at T3 (Figure 3K). Similarly, the GSH contents had an increasing trend (*p* < 0.1) at T2 and were significantly higher (*p* < 0.05) at T3 in the ST + TA group compared with the ST group (Figure 3L). While the elevation (*p* < 0.1) in MDA levels occurred at T2 in both the groups (Figure 3M). The results indicated that TA may relieve oxidative stress and inflammatory response by enhancing enzymatic and non-enzymatic anti-oxidant systems as well as regulating the secretion of anti- or pro-inflammatory cytokines.

### Effect of Tannic Acid on Fecal Microbiota

**FIGURE 4:**
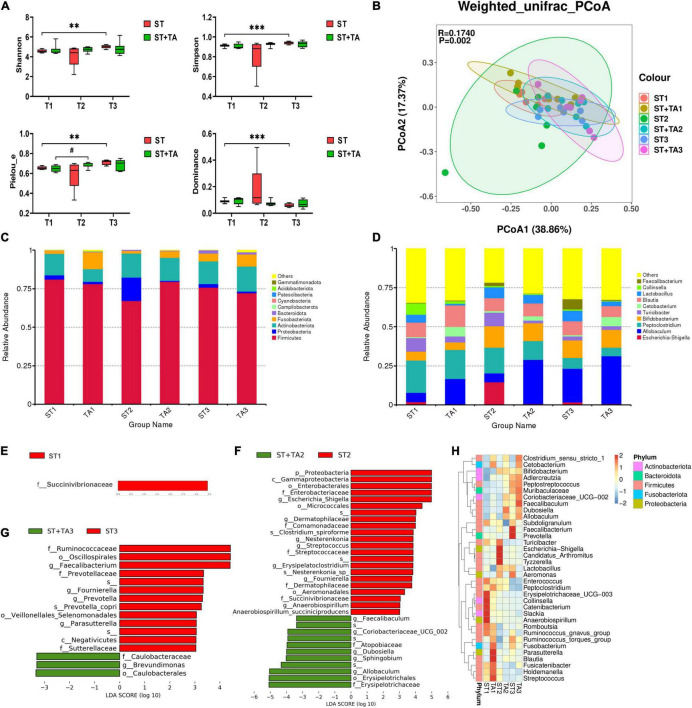
Effect of TA on gut microbiota composition and structure in dogs. α-diversity analysis (Shannon, Simpson, Pielou_e, and Dominance) **(A)**, principal coordinate analysis (PCoA) based on weighted UniFrac distances **(B)**, histogram of abundance distribution at the phylum level **(C)** and genus level **(D)**, the LEfSe analysis among groups **(E–G)**, and hierarchical clustering **(H)**. Data are presented as mean ± SE (*n* = 6 or 7). ^**^p < 0.01 and ^***^p < 0.001 represent the difference of each group at T1–T3 calculated by RM-ANOVA. ^#^*p* < 0.10 represents the difference in tendency of each group at T1–T3 calculated by RM-ANOVA. The *p*-values of β-diversity index in the PCoA calculated by the Wilcoxon rank sum test. T1, day 7 before transportation; T2, day 8 after transportation; T3, day 14 after transportation.

The Venn diagram revealed that both groups had the fewest ASVs at T2, whereas the livable environment and TA supplementation had increased ASVs at T3 (Supplementary Figure 1). Relative to the ST group, puppies fed TA had more specific ASVs. Additionally, Good’s coverage in all samples was 100%. As shown in Figure 4A, Shannon, Simpson, and Pielou_e indexes in the ST group were higher (*p* < 0.01) at T3 compared with T1; and puppies fed TA had an increasing trend (*p* < 0.1) of Pielou_e index at T2 compared with T1; while lower (*p* < 0.001) Dominance index was found in the ST group at T3 compared with T1. No significant difference was found in Observed_species and Chao1 (Supplementary Figure 2). Overall, the livable environment and TA supplementation had a positive impact on the bacteria diversity in dogs. The PCoA was used to examine the similarity of gut microbial structure. The PcoA plots based on weighted UniFrac distances revealed distinct separation between the groups (*p* < 0.01, Figure 4B), indicating that environment and TA supplementation could influence gut microbiota composition and diversity in dogs.

As shown in Figure 4C, the top five phyla were Firmicutes, Actinobacteriota, Fusobacterota, Proteobacteria, and Bacteroidota, accounting for about 90% of the total bacteria. The ST + TA group had a higher level of Fusobacterota at T1 and a lower level of Proteobacteria at T2 than those in the ST group. At the genus level, the most abundant genera were *Allobaculum*, *Bifidobacterium*, *Peptoclostridium*, *Blautia*, *Lactobacillus*, *Turicibacter*, *Cetobacterium*, and *Escherichia-Shigella* (Figure 4D). Among them, the ST group had the highest *Escherichia-Shigella* and the lowest *Allobaculum* at T2. The LefSe (LDA > 3) analysis was further employed for the identification of potential biomarkers. At T1, Succinivibrionaceae significantly enriched in the ST group (Figure 4E). At T2, *Escherichia-Shigella, Dermatophilaceae*, *Nesterenkonia*, *Streptococcus*, *Erysipelatoclostridium*, *Fournierella*, and *Anaerobiospirillum* were remarkably enriched in the ST group, and *Allobaculum*, *Sphingobium*, *Dubosiella*, *Coriobacteriaceae_UCG-002*, and *Faecalibaculum* were remarkably enriched in the ST + TA group (Figure 4F). At T3, *Faecalibacterium*, *Fournierella*, *Prevotella*, and *Parasutterella* showed significant enrichments in the ST group, and *Brevundimonas* showed significant enrichment in the ST + TA group (Figure 4G). Based on the results of the 35 most-abundant bacteria genera, we also constructed a clustered heatmap, which showed similar results with the LefSe (Figure 4H). Collectively, these results indicated that environmental stress caused the differences in microbial composition, while intestinal microbiota developed in a more favorable direction when dogs were transported to a livable environment and added TA.

**FIGURE 5:**
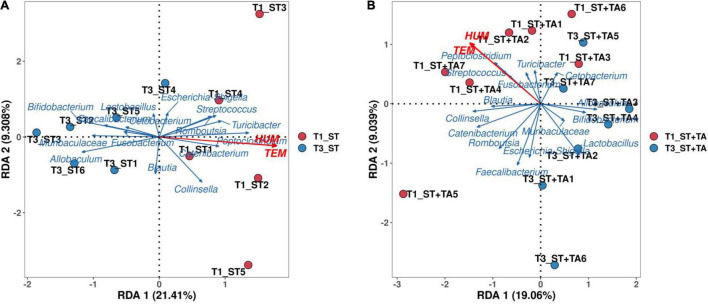
Redundancy analysis (RDA) of environmental factors and the microbial community at T1 and T3. RDA in the ST group at T1 and T3 **(A)**, RDA in the ST + TA group at T1 and T3 **(B)**. T1, day 7 before transportation; T3, day 14 after transportation.

A detrended correspondence analysis (DCA) was performed to select the appropriate ordination analysis method (gradient lengths were less than 3). The RDA method was applied to analyze the complex associations between microbiota composition and environmental factors (temperature and humidity). We selected the top 15 genera in the relative abundance and environmental factors for the RDA analysis. RDA axes 1 and 2 accounted for 21.41 and 9.31% in the ST group at T1 and T3 (Figure 5A), and 19.06 and 9.04% in the ST + TA group at T1 and T3 (Figure 5B), respectively, of the total variation. The angle between temperature (TEM) and humidity (HUM) was acute, thus, they had a positive correlation. The differences in the dominant bacterial genera were associated with the differences in TEM and HUM. TEM and HUM had positive associations with *Streptococcus*, *Peptoclostridium*, *Turicibacter*, *Catenibacterium*, and *Collinsella* at T1, and negative associations with *Bifidobacterium*, *Lactobacillus*, *Allobaculum*, and *Muribaculaceae* at T3.

### Effect of Tannic Acid on Fecal Short-Chain Fatty Acids and Branched-Chain Fatty Acids

**FIGURE 6:**
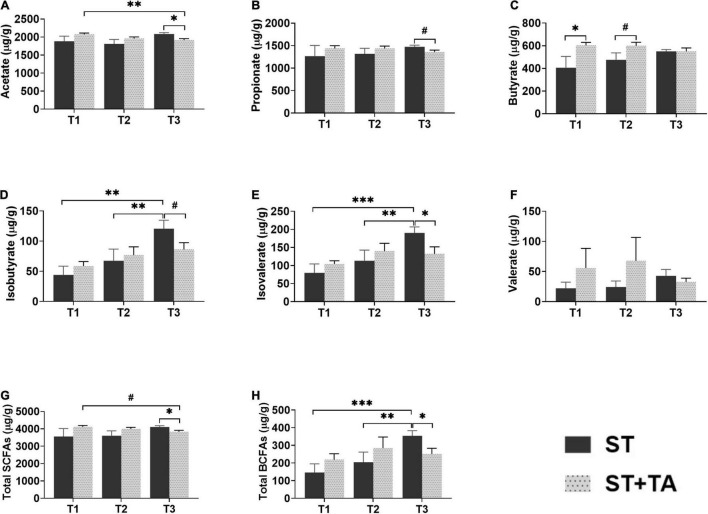
Effect of TA on acetate **(A)**, propionate **(B)**, butyrate **(C)**, isobutyrate **(D)**, isovalerate **(E)**, valerate **(F)**, total SCFAs **(G)**, and total BCFAs **(H)** in dogs. Data are presented as mean ± SE (*n* = 6 or 7). *p < 0.05, ^**^p < 0.01, and ^***^p < 0.001 represent the difference calculated by Student’s *t*-test between the ST and ST + TA groups or the difference of each group at T1–T3 calculated by RM-ANOVA; ^#^*p* < 0.10 represents the difference in tendency calculated by Student’s *t*-test between the ST and ST + TA groups or the difference in tendency of each group at T1–T3 calculated by RM-ANOVA. T1, day 7 before transportation; T2, day 8 after transportation; T3, day 14 after transportation. Total SCFAs (short-chain fatty acids) = acetate + propionate + butyrate; Total BCFAs (branched-chain fatty acids) = isobutyrate + isovalerate + valerate.

The SCFAs serve as the important energy source for the intestinal epithelium, and BCFAs are metabolites that result from protein fermentation. Fecal acetate and propionate content at T3 in the ST + TA group were less (*p* < 0.05) than that in the ST group, and the ST + TA group had lower (*p* < 0.01) fecal acetate at T3 than that at T1 (Figures 6A,B). As a result, higher (*p* < 0.05) total SCFAs were observed at T3 in the ST group (Figure 6G). However, dogs fed TA had a significant increase (*p* < 0.05) in butyrate at T1 and had an increasing trend in butyrate (*p* < 0.1) at T2 compared with the ST group (Figure 6C). Furthermore, the ST group showed an evident increase (*p* < 0.01) of fecal isobutyrate and isovalerate at T3 compared with T1 and T2, while supplementation with TA significantly decreased (*p* < 0.05) fecal isobutyrate and isovalerate at T3. Similar alterations (*p* < 0.05) also occurred in the total BCFAs level (Figures 6D,E,H). No significance was observed in fecal valerate (Figure 6F).

### Effect of Tannic Acid on Serum Metabolome

To investigate the metabolic regulation in TA-treated dogs, serum metabolites were analyzed using UPLC-Orbitrap-MS/MS. A total of 147 metabolites were detected in each group. The PCA analysis found that the ST and ST + TA groups had a partial separation at T2, while there was no obvious separation between the two groups at T1 and T3 (Supplementary Figures 3A–C). In the clustering heat map, the accumulation of serum metabolites displayed a clear variation in terms of the pattern of metabolite abundance at different time points (Supplementary Figure 4), indicating that environmental stress and adding TA can result in changing serum metabolic profiles in dogs.

**FIGURE 7:**
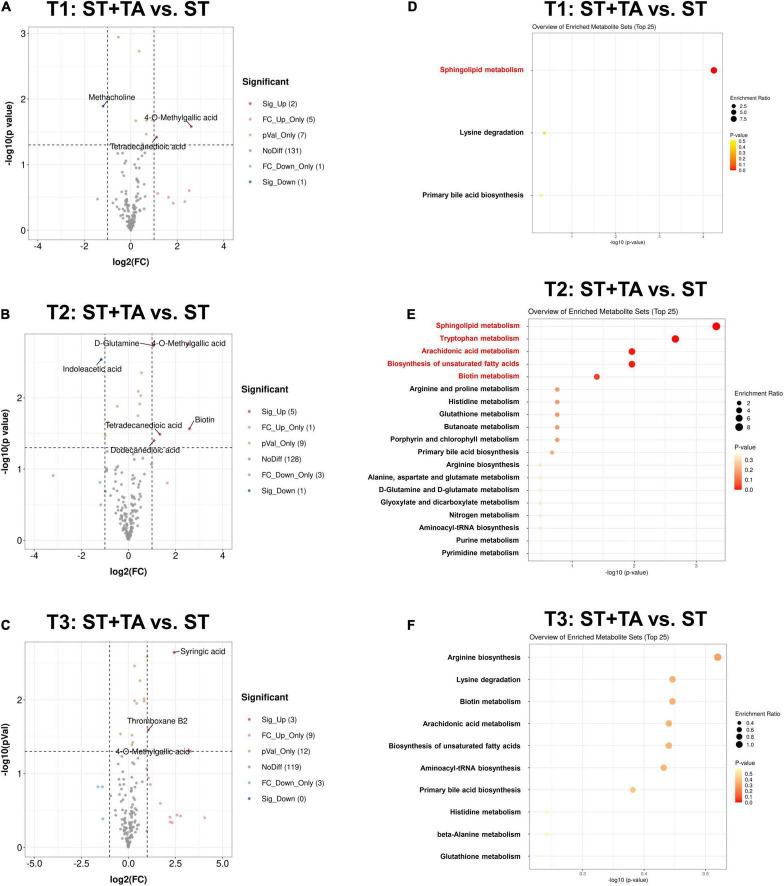
Effect of TA on serum metabolome in dogs. Volcano plot **(A–C)** and enrichment analysis of metabolic pathways **(D–F)**. The red font indicates significantly different metabolic pathways (*p* < 0.05). T1, day 7 before transportation; T2, day 8 after transportation; T3, day 14 after transportation.

Next, differential metabolites were screened with FC > 2 (or < 0.5) and *p* < 0.05, and 15, 18, and 27 serum metabolites at T1, T2, and T3 were significantly changed between the two groups (Supplementary Table 1). The volcano plot showed that the primary significant metabolites were 4-*O*-Methylgallic acid, tetradecanedioic acid, and methacholine at T1, 4-*O*-Methylgallic acid, biotin, indoleacetic acid, dodecanedioic acid, tetradecanedioic acid, and D-glutamine at T2, and 4-*O*-Methylgallic acid, syringic acid, and thromboxane B2 at T3 (Figures 7A–C). As shown in Figures 7D–F and Table 1, dogs fed TA mainly influenced sphingolipid metabolism at T1, and TA changed sphingolipid metabolism, tryptophan metabolism, arachidonic acid metabolism, biosynthesis of unsaturated fatty acids, and biotin metabolism at T2. No difference was observed at T3.

**TABLE 1:** Significant metabolic pathways with its matched differential metabolites at varying time points.

Time point ^a^	Metabolite	Metabolic pathway	Metabolism	Trend (ST + TA vs. ST)
T1	Phytosphingosine	Sphingolipid metabolism	Lipid metabolism	Down
T2	Phytosphingosine	Sphingolipid metabolism	Lipid metabolism	Down
T2	Indoleacetic acid	Tryptophan metabolism	Amino acid metabolism	Down
T2	Arachidonic acid	Arachidonic acid metabolism	Lipid metabolism	Down
T2	Arachidonic acid	Biosynthesis of unsaturated fatty acids	Lipid metabolism	Down
T2	Biotin	Biotin metabolism	Metabolism of cofactors and vitamins	Up

*^a^T1, day 7 before transportation; T2, day 8 after transportation.*

### Correlation Analysis Between Fecal Bacteria at the Genus Level and Metabolites

**FIGURE 8:**
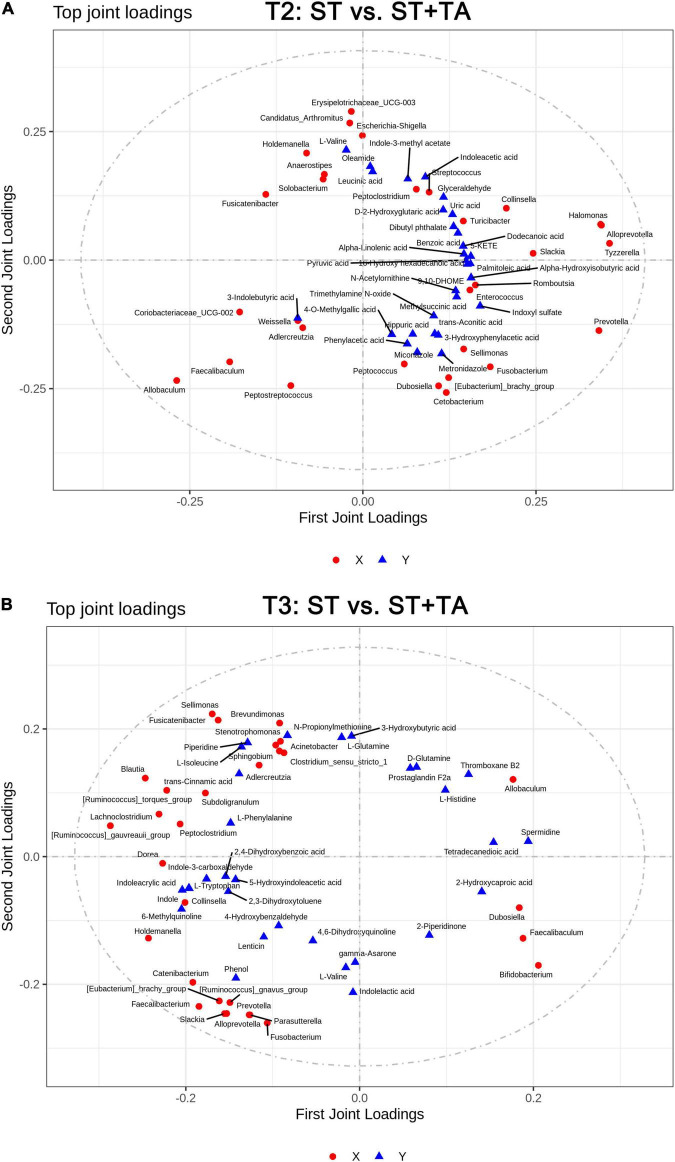
Two-way orthogonal partial least squares (O2PLS) analysis of significant features identified from the microbiome and metabolomics analysis. Microbiome-metabolomics correlation loading plots between the ST and ST + TA groups at T2 **(A)** and T3 **(B)**. Red circle indicates bacteria at the genus level, and blue triangle indicates metabolite. The greater the absolute value in the coordinate, the greater the degree of association of this microbiota/metabolite with another omics. T2, day 8 after transportation; T3, day 14 after transportation.

The O2PLS method was performed to analyze the association between microbiota and metabolites. It was shown that R2X and R2Y of the model were 0.486 and 0.633 at T2, and 0.791 and 0.509 at T3, indicating that the O2PLS method was well suited for analysis and prediction. As shown in Figures 8A,B, the top-ranking 30 loading values, the contribution degree of the variable (microbiota/metabolite) to the difference between groups, were displayed in the microbiome–metabolomics correlation loading plots (all loading values are listed in Supplementary Table 2). The O2PLS as an initial screen for microbiome and metabolomic correlation analyses to avoid false-positive associations as much as possible. From these figures, we obtained microbiota and metabolites with a high correlation, which can provide a reference for subsequent correlation analysis.

**FIGURE 9:**
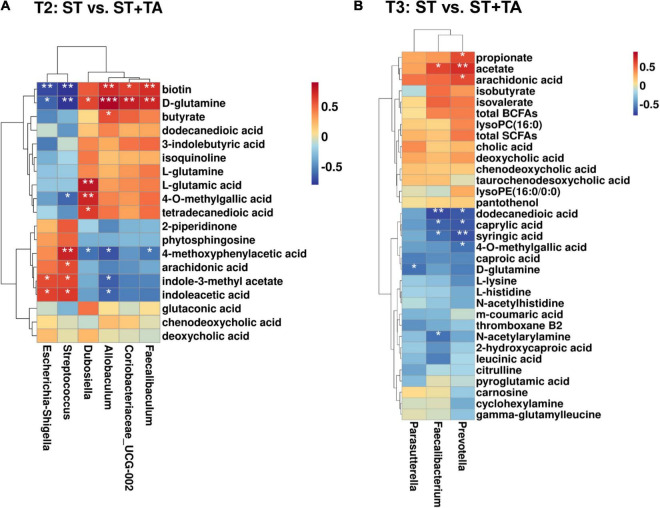
Correlation heatmap between differential metabolites and fecal bacteria (at the genus level) at T2 **(A)** and T3 **(B)**. The symbol (*) indicates a significant correlation between serum metabolites and fecal bacteria (*p < 0.05, **p < 0.01, and ***p < 0.001). Red color indicates a positive correlation, and blue color indicates a negative correlation. T2, day 8 after transportation; T3, day 14 after transportation.

Spearman correlation between differential serum metabolites and bacteria genera with relative abundance greater than 0.1% was displayed in a heatmap. At T2, *Escherichia-Shigella* and *Streptococcus* had significant positive associations with indole-3-methyl acetate and indoleacetic acid and negative associations with biotin and D-glutamine (Figure 9A); and *Streptococcus* had significant positive associations with arachidonic acid and 4-methoxyphenylacetic acid and a negative association with 4-*O*-methylgallic acid. Conversely, *Allobaculum*, *Coriobacteriaceae_UCG-002*, and *Faecalibaculum* had opposite associations with *Escherichia-Shigella* and *Streptococcus*, and *Allobaculum* had a significant positive association with butyrate. Also, *Dubosiella* was positively correlated with D-glutamine, L-glutamine, 4-*O*-methylgallic acid, and tetradecanedioic acid and negatively correlated with 4-methoxyphenylacetic acid. At T3, a significant negative association between *Parasutterella* and D-glutamine was found (Figure 9B). *Prevotella* and *Faecalibacterium* had significant positive associations with acetate and negative associations with dodecanedioic acid, caproic acid, and syringic acid; *Faecalibacterium* had a negative association with N-acetylarylamine; and *Prevotella* had significant positive associations with propionate and arachidonic acid and a negative association with 4-*O*-methylgallic acid.

## Discussion

In this study, we selected beagle dogs as the animal model to explore the anti-stress effects of gallnut TA. Similar to our previous study in puppies under environmental stress (40), the body condition, diarrhea rate, serum biochemistry, hematology, HSP-70, hormone, inflammatory response, oxidative stress, fecal microbiota, and serum metabolic profiles were significantly different among various environments, indicating that puppies under high temperature and high humidity remained in a state of stress. Our results suggest that dietary supplementation of 2.5 g/kg TA improves the health of dogs under stress. Recently, several studies have also reported the applications and effects of TA in weaned piglets and mice (42, 43, 52, 53), supporting the anti-diarrheal, anti-oxidative, anti-inflammatory, and anti-microbial activities of TA without affecting production performance. Consistent with previous studies, dogs showed an evident decrease in BCS after transportation because of vomiting and diarrhea. After the supplementation of TA, dogs could maintain a good body condition, normal fecal shape, and lower incidence of diarrhea *via* the regulation of gut microbiota. A few previous studies found that the stressful environment had significant effects on serum biochemistry and blood routine in animals, including causing injury to the liver (TP, ALB, AST, ALT, and ALP), kidney (Cr, BUN, and uric acid), myocardium (CK and LDH), and dyslipidemia (TC, TG, LDL-C, and HDL-C) (54–56), as well as the upregulation of peripheral innate immune cells (granulocyte, lymphocyte, and monocytes) (57). Similarly, the present study provided evidence that a stressful environment caused abnormal liver function and myocardial injury in dogs, while the injury was alleviated when transported to a new livable site, and TA had a further protective effect on myocardial injury (decreasing LDH) in dogs. In addition, decreased levels of WBC, NE, HGB, MCH, and MCHC were observed in dogs when transported to a livable environment, indicating that both environmental change and TA supplementation help to enhance immunity. In brief, TA can relieve organ damage and inflammatory response through modulating serum metabolites and immune cells.

Serum COR is considered as a biomarker for stress evaluation in dogs (58, 59). The COR is the primary GC secreted by the adrenal gland in response to ACTH stimulation. ACTH stimulates the secretion of COR from the adrenal cortex into the peripheral bloodstream (60, 61). This study found a significant increase in serum COR concentration in dogs after transportation, suggesting that environmental stresses activated the HPA axis, and triggered stress responses in dogs. As expected, ACTH and GC displayed a similar alteration trend with COR. Though dogs fed TA had an increase in COR, GC, and ACTH secretion, TA still had the potential to alleviate stress relative to an extremely significant elevation in the ST group. The HSP-70, a kind of stress-induced protein, protects cells against stresses (62). In the present study, the upregulation of HSP-70 in stressed dogs after transportation might be an adaptation mechanism in response to environmental stress (63). Studies *in vivo* and *in vitro* revealed dietary polyphenolic compounds (e.g., curcumin, phloretin, and chlorogenic acid) could modulate the HSP-70 expression (64–66). Thus, TA might inhibit the expression of HSP-70 to protect dogs from stress-induced injury.

Oxidative stress may be caused by an imbalance between reactive oxygen species and the anti-oxidant system, which would result in generating inflammation (67). Therefore, oxidative stress and inflammation are closely linked (68). A recent study demonstrated that goats that were subject to transportation stress had better stress-resistant, anti-oxidant, and anti-inflammatory capacities when fed diets containing condensed tannins (69). Both condensed and hydrolyzable tannins belong to tannins with different molecular structures (70). Overall, as a family of hydrolyzable tannins, gallnut TA displayed strong anti-oxidative and anti-inflammatory capacities in dogs after transportation. TA could normalize or enhance anti-oxidant systems, including non-enzymatic systems (GSH) and enzymatic systems (SOD and GSH-Px), after transportation. Constantly elevated T-AOC level indicated the total anti-oxidant level of enzymatic and non-enzymatic systems in the ST + TA group. Therefore, an imbalance between the formation of reactive oxygen species and the anti-oxidant defense increased the secretion of pro-inflammatory cytokines (TNF-α, IL-2, and IL-6) and decreased the secretion of anti-inflammatory cytokine IL-4 due to transportation and a varying environment. Likewise, TA exhibited a very good anti-inflammatory property.

Environmental and physical stresses can regulate the gut microbiota (71), which influences the host stress response. Gut microbiota thereby serves as an important mediator for host health (1, 72). Substantial studies toward polyphenols have reported that polyphenols are capable of acting as prebiotics to promote the growth of beneficial gut microbiota (73–75). Five predominant bacterial phyla are identified in the canine gastrointestinal tract: Firmicutes, Fusobacteria, Bacteroidetes, Proteobacteria, and Actinobacteria (76, 77). The dominant phyla in the feces in this work were also consistent with prior studies. Moreover, the gut microbial community was greatly changed after transportation. We observed the differences in microbial composition (Shannon, Simpson, Pielou_e, and Dominance indexes) and microbial community structure (PCoA), indicating that the stressful environment had a negative effect on the composition and structural diversity of gut microbiota in dogs. There were a few significant differences in gut microbiota between the groups before transportation (T1). At T2, multiple environmental stressors promoted the growth of pathogenic bacteria (*Escherichia-Shigella and Streptococcus*) (78, 79), thereby leading to diarrhea and inflammatory cytokine secretion. Meanwhile, *Allobaculum*, *Dubosiella*, *Coriobacteriaceae_UCG-002*, and *Faecalibaculum* highly enriched in dogs fed TA. Similar to our association analysis results, *Allobaculum*, *Dubosiella*, and *Faecalibaculum* had positive associations with the production of butyrate in previous reports (80–85). Butyrate has been shown to have critical roles in the maintenance of intestinal homeostasis, consequently leading to the alleviation of diarrhea (86). Moreover, *Faecalibaculum* and *Coriobacteriaceae_UCG-002* showed significantly negative correlations with ALT, AST, AKP, and MDA levels (87). At T3, SCFAs-producing bacteria *Prevotella*, *Faecalibacterium*, and *Parasutterella* (88–91) showed significant enrichments in the ST group, revealing that a livable environment in dogs caused the production of SCFAs (acetate, propionate, and total SCFAs). Meanwhile, supplementation with TA decreased fecal BCFAs (isobutyrate, isovalerate, and total BCFAs), which are harmful putrefactive components produced during the fermentation of branched-chain amino acids (92). In addition, the microbial community structure in dogs can rapidly change in response to altered environmental conditions (40). Likewise, similar results were obtained in our study. The RDA analysis revealed that the high TEM (29^°^C) and HUM (96%) promoted intestinal pathogen development, and the suitable TEM (23^°^C) and HUM (70%) stimulated the growth of intestinal beneficial bacteria in dogs. Although puppies fed gallic acid also reached similar inhibitory and promoting effects on pathogenic bacteria (Proteobacteria, *Escherichia–Shigella*, and *Clostridium_sensu_stricto_1*) and beneficial bacteria (Firmicutes, *Faecalibaculum*, and *Lactobacillus*) (40), feeding TA or gallic acid affect the different types of intestinal bacteria in puppies, indicating that TA is not fully hydrolyzed to gallic acid to influence gut microbiota.

Gut microbiota contributes to host metabolism, protects against pathogens, and modulates the immune system, thereby affecting host physiological functions (76). Thus, the metabolomics analysis based on UPLC-Orbitrap-MS/MS was adopted to explore the significant changes of serum metabolites to identify the related metabolic pathways in beagle dogs. First, a high level of serum 4-*O*-methylgallic acid resulting from the hydrolyzed TA was observed in the ST + TA group at varying time points (93), which was consistent with a single addition of gallic acid on stressful puppies (40). According to correlation analysis, we found that 4-*O*-methylgallic acid had positive associations with *Dubosiella*. Hence, we speculated that it was able to produce tannases to catabolize TA, generating 4-*O*-methylgallic acid in the serum. Further investigation is needed to verify this hypothesis. Previous studies showed that environmental changes resulted in psychological alterations, and thereby may affect metabolism (40, 94). In this study, we also observed that serum phytosphingosine (belonging to the sphingolipid metabolism) content was downregulated at T1 and T2 when supplementation with TA, indicating that the stress stimulated sphingolipid synthesis and metabolism during transportation. A study also found that chemical stress (acrylamide contact) could stimulate serum phytosphingosine production, which might be associated with the nervous system symptoms and the abnormity of the biochemical indexes of AST and ALT (95). Interestingly, in a model of aging induced by D-galactose, an obvious decrease of phytosphingosine was observed under the green tea polyphenol treatment (96). As a typical accumulated uremic toxin, indoleacetic acid has been associated with the oxidative stress and inflammation response, which play a role in the progression of chronic kidney disease and the development of complications (97, 98). Arachidonic acid is a precursor to several pro-inflammatory/pro-aggregatory mediators (prostaglandins, thromboxanes, and leukotrienes) (99, 100). Current results showed that dogs fed with TA downregulated indoleacetic acid (belonging to the tryptophan metabolism) and arachidonic acid (belonging to the arachidonic acid metabolism and biosynthesis of unsaturated fatty acids) levels at T2. Instead, TA increased serum biotin (belonging to the biotin metabolism) concentration at T2. Biotin, a water-soluble vitamin, serves as a coenzyme for carboxylases in humans, and biotin deficiency influences cell proliferation, immune function, and fetal development (101–103). Thus, the dietary supplementation of TA relieves oxidative stress and inflammation induced by transportation and environmental changes *via* the regulation of host metabolism. A study on gallic acid alleviating stress in puppies found that gallic acid reversed the abnormalities of host amino acid metabolism, lipid metabolism, carbohydrate metabolism, and nucleotide metabolism (40), but its specific metabolic pathways are different from TA. One reason may be that TA was not fully hydrolyzed to gallic acid, and uncertain time for hydrolysis of TA to gallic acid may be also an important reason.

Additionally, the correlation analysis between serum metabolites and fecal bacteria indicated that (1) both serum indoleacetic acid and arachidonic acid were positively correlated with *Streptococcus*; (2) serum indoleacetic acid was negatively correlated with *Allobaculum*; and (3) serum biotin was positively correlated with *Allobaculum*, *Coriobacteriaceae_UCG-002*, and *Faecalibaculum* and negatively correlated with *Escherichia-Shigella* and *Streptococcus*. Further analysis can focus on the calculation of omics-explainability (104), which was utilized to estimate the contribution rate of omics to phenotype. We suggest that phytosphingosine, indoleacetic acid, arachidonic acid, and biotin could serve as potential biomarkers of the environmental stress response. Nonetheless, the relationship between intestinal microbiota and its metabolites needs further investigation. In short, dietary TA may be a potential prebiotic for the prevention and treatment of metabolic disorders by targeting intestinal microbiota.

## Conclusion

In conclusion, we found that dietary supplementation of TA at 2.5 g/kg relieved environmental stress-induced diarrheal symptoms, oxidative stress, and inflammation in dogs. Fecal microbiota detected by high-throughput 16S rRNA gene sequencing revealed that TA stimulated the growth of beneficial bacteria *Allobaculum*, *Dubosiella*, *Coriobacteriaceae_UCG-002*, and *Faecalibaculum* and suppressed the growth of pathogenic bacteria *Escherichia-Shigella* and *Streptococcus*, thereby promoting intestinal health by increasing butyrate levels in dogs after transportation for 1 day. In addition, the relative abundance of *Faecalibacterium*, *Prevotella*, and *Parasutterella*, as well as the consequent SCFAs (acetate, propionate, and total SCFAs) increased in dogs when transported from a stressful environment to a livable environment for 7 days, which played a critical role in the maintenance of intestinal homeostasis. However, the relationship between SCFAs and intestinal microbiota in dogs needs to be further explored. Serum metabolomics further showed that phytosphingosine, indoleacetic acid, arachidonic acid, and biotin, related to sphingolipid metabolism, tryptophan metabolism, arachidonic acid metabolism and biosynthesis of unsaturated fatty acids, and biotin metabolism, respectively, could serve as potential biomarkers of the environmental stress response. Spearman’s correlation analysis further showed the tight relationships between the four potential serum biomarkers (phytosphingosine, indoleacetic acid, arachidonic acid, and biotin) and differential bacteria (*Allobaculum*, *Coriobacteriaceae_UCG-002*, *Faecalibaculum*, *Escherichia-Shigella*, and *Streptococcus*). However, these relationships require further verification. In all, gallnut TA may be a potential prebiotic for the prevention and treatment of metabolic disorders by targeting intestinal microbiota.

## Data Availability Statement

The datasets presented in this study can be found in online repositories. The names of the repository/repositories and accession number(s) can be found below: https://www.ncbi.nlm.nih.gov/bioproject/PRJNA792485.

## Ethics Statement

The animal study was reviewed and approved by the Experimental Animal Ethics Committee of South China Agricultural University.

## Author Contributions

KY generated ideas, carried out the experiment, and wrote the initial manuscript. BD, SH, and QL designed the study and revised the manuscript. SJ participated in the data analysis and contributed to the draft of this manuscript. KY, CW, DG, PL, JW, and TK detected the samples. All authors contributed to the article and approved the submitted version.

## Conflict of Interest

The authors declare that the research was conducted in the absence of any commercial or financial relationships that could be construed as a potential conflict of interest.

## Publisher’s Note

All claims expressed in this article are solely those of the authors and do not necessarily represent those of their affiliated organizations, or those of the publisher, the editors and the reviewers. Any product that may be evaluated in this article, or claim that may be made by its manufacturer, is not guaranteed or endorsed by the publisher.

## Footnotes

1
http://bioinformatics.psb.ugent.be/webtools/Venn/

2
https://www.metaboanalyst.ca
