# Supplementary material for: Gallnut Tannic Acid Exerts Anti-stress Effects on Stress-Induced Inflammatory Response, Dysbiotic Gut Microbiota, and Alterations of Serum Metabolic Profile in Beagle Dogs
Source: Front Nutr. 2022 Apr 27;9:847966. doi: 10.3389/fnut.2022.847966 (PMC9094144; doi:10.3389/fnut.2022.847966)
Supplement: Supplementary file 1 [file Data_Sheet_1.docx]

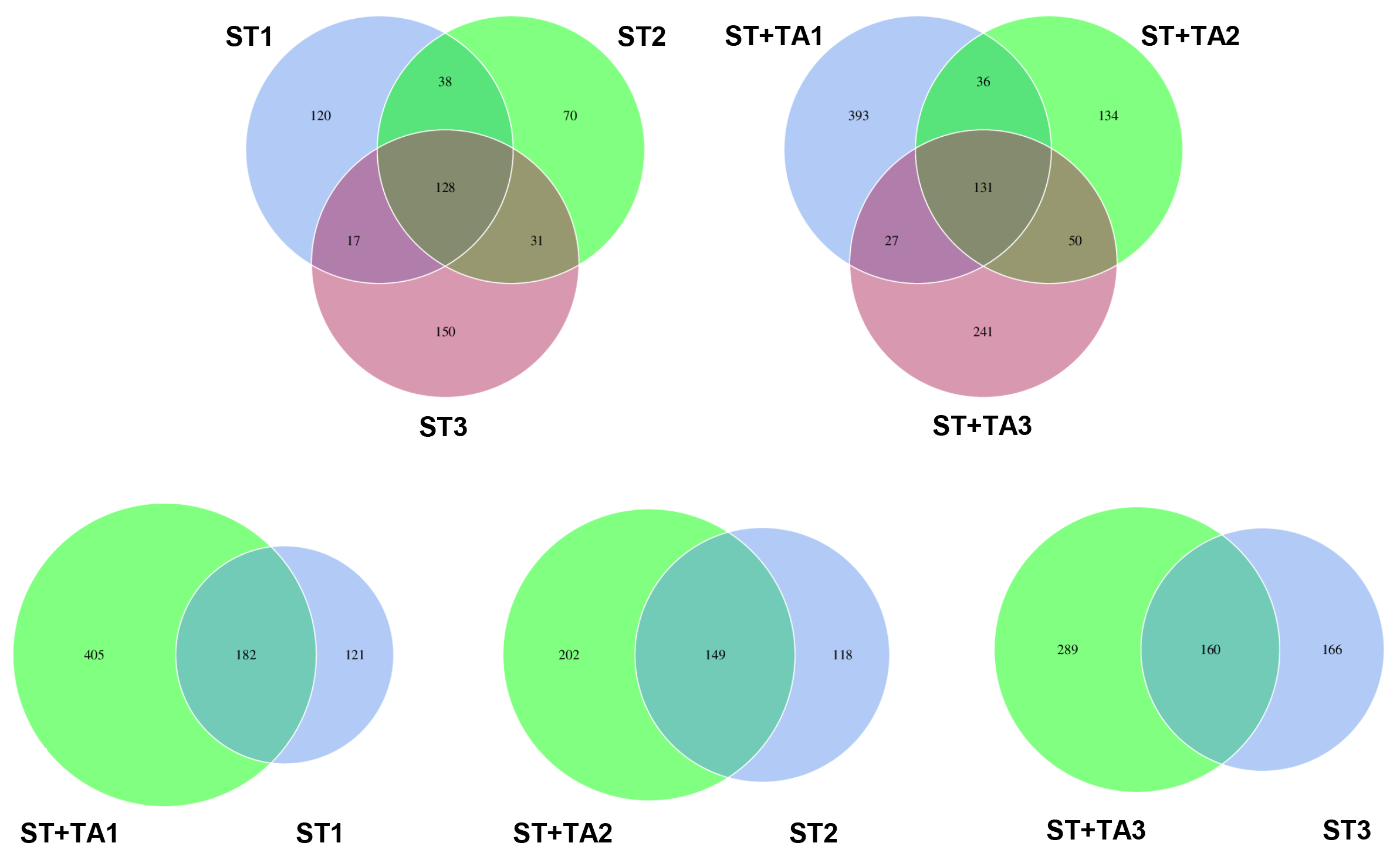


**FIGURE S1 |** Venn diagrams based on ASVs. T1, day 7 before transportation; T2, day 8 after transportation; T3, day 14 after transportation.


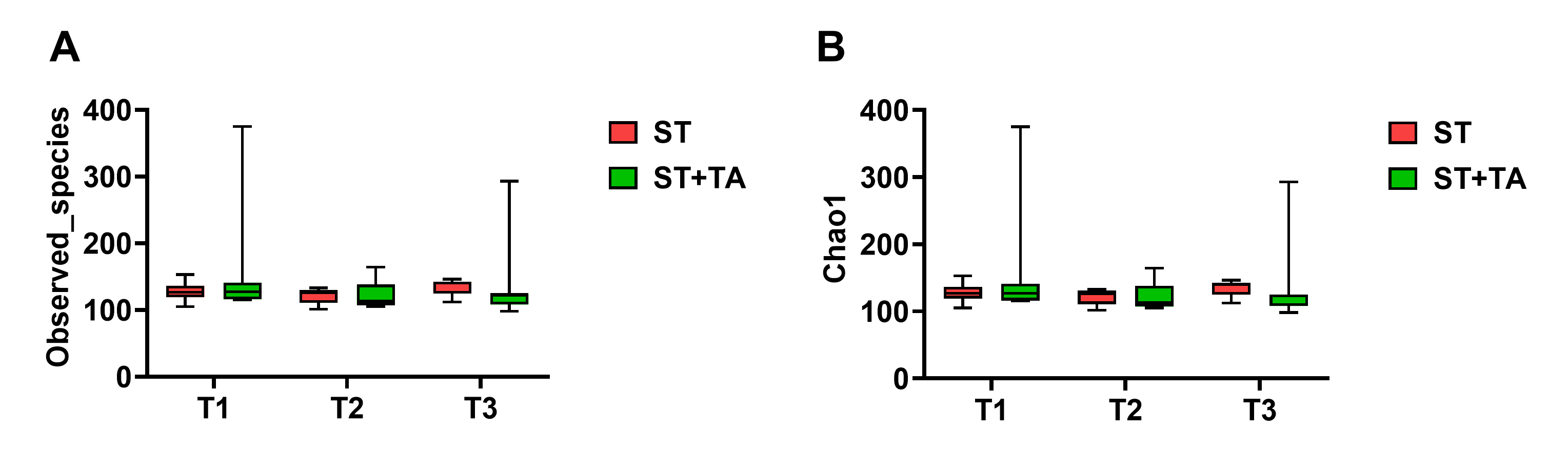


**FIGURE S2 |** Effect of tannic acid (TA) on α-diversity of gut microbiota in puppies including Observed_species (**A**) and Chao1 (**B**). T1, day 7 before transportation; T2, day 8 after transportation; T3, day 14 after transportation.





**FIGURE S3 |** Score plots from the PCA model between the ST and ST+TA groups at T1 (**A**), T2 (**B**), and T3 (**C**). T1, day 7 before transportation; T2, day 8 after transportation; T3, day 14 after transportation.


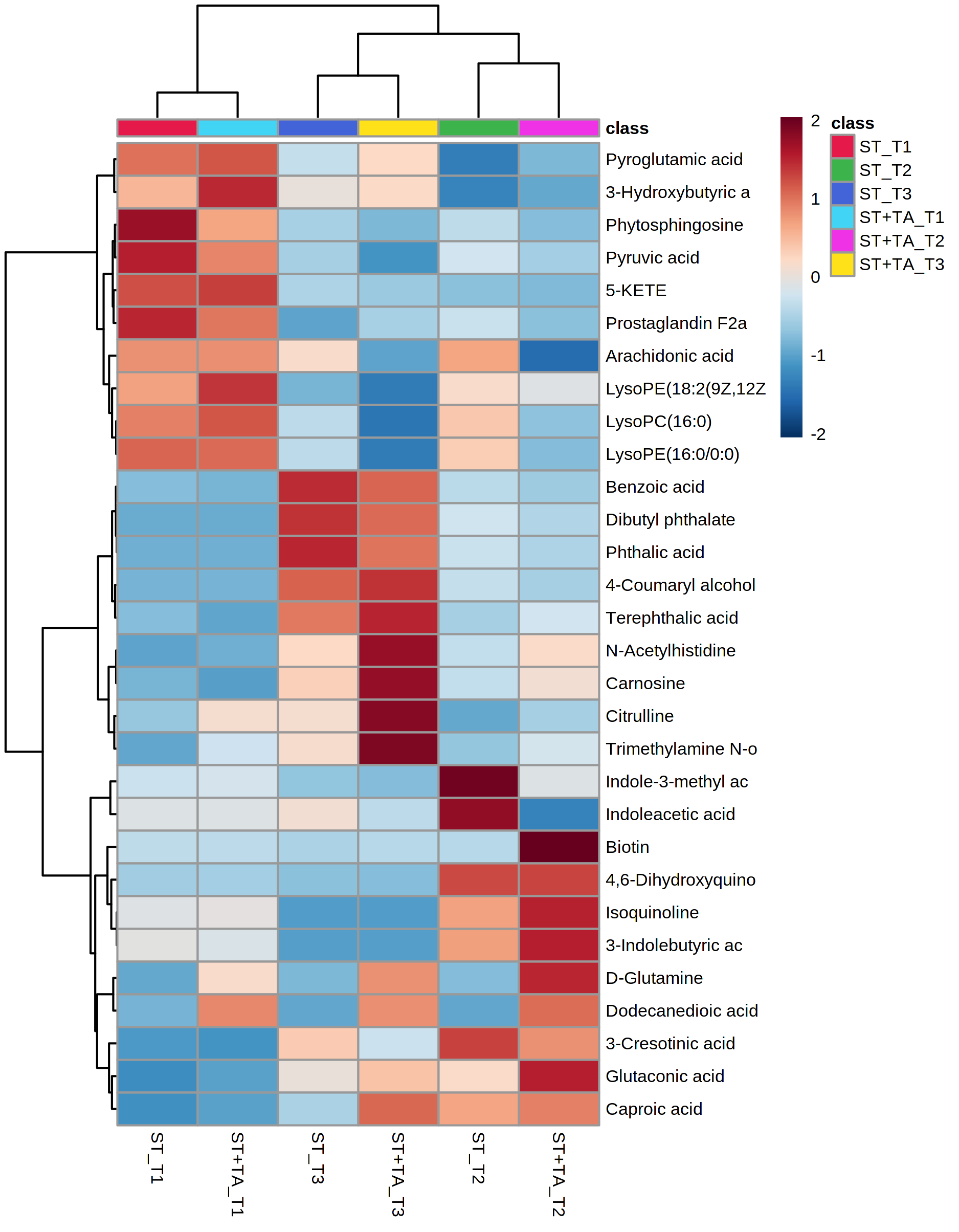


**FIGURE S4 |** Clustering heat map of serum metabolites. T1, day 7 before transportation; T2, day 8 after transportation; T3, day 14 after transportation.

**TABLE S1 |** Significant metabolites and metabolic pathways at varying time points.

| Time point ^1^ | Metabolite | Fold change ^2^ | *p*-value ^3^ | Trend  (ST+TA vs. ST) |
| --- | --- | --- | --- | --- |
| T1 | 4-O-Methylgallic acid | **6.0825** | **0.026232** | up |
| T1 | Methacholine | **0.43729** | **0.012846** | down |
| T1 | Tetradecanedioic acid | **2.1641** | **0.037987** | up |
| T1 | Deoxycholic acid | **5.0061** | 0.36615 | up |
| T1 | Chenodeoxycholic acid | **3.5485** | 0.38797 | up |
| T1 | m-Coumaric acid | **3.0792** | 0.31434 | up |
| T1 | 4-Methoxyphenylacetic acid | **0.37037** | 0.33516 | down |
| T1 | gamma-Asarone | **2.2301** | 0.27388 | up |
| T1 | Phytosphingosine | 1.4438 | **0.001137** | down |
| T1 | L-Carnitine | 0.77767 | **0.001863** | up |
| T1 | Dodecanedioic acid | 0.52454 | **0.02102** | up |
| T1 | D-Glutamine | 0.6278 | **0.021097** | up |
| T1 | (±)-2-(1-Methylpropyl)-4,6-dinitrophenol | 0.86261 | **0.02147** | up |
| T1 | Hexadecanedioic acid | 0.63086 | **0.034195** | up |
| T1 | Octadecanedioic acid | 0.70348 | **0.047862** | up |
| T2 | Biotin | **6.013** | **0.027245** | up |
| T2 | 4-O-Methylgallic acid | **5.8089** | **0.0017709** | up |
| T2 | Tetradecanedioic acid | **2.5236** | **0.032535** | up |
| T2 | Indoleacetic acid | **0.44878** | **0.0028853** | down |
| T2 | Dodecanedioic acid | **2.1267** | **0.039687** | up |
| T2 | D-Glutamine | **2.0829** | **0.0018491** | up |
| T2 | 4-Methoxyphenylacetic acid | **0.10918** | 0.12364 | down |
| T2 | Deoxycholic acid | **0.43226** | 0.15404 | down |
| T2 | Chenodeoxycholic acid | **0.44317** | 0.31794 | down |
| T2 | L-Glutamic acid | 1.4679 | **0.0044534** | up |
| T2 | Isoquinoline | 1.336 | **0.0080861** | up |
| T2 | Glutaconic acid | 1.4391 | **0.0092981** | up |
| T2 | L-Glutamine | 1.3901 | **0.012175** | up |
| T2 | Phytosphingosine | 0.72204 | **0.013195** | down |
| T2 | 3-Indolebutyric acid | 1.3256 | **0.017854** | up |
| T2 | Indole-3-methyl acetate | 0.54434 | **0.021907** | down |
| T2 | Arachidonic acid | 0.57067 | **0.024531** | down |
| T2 | 2-Piperidinone | 0.50489 | **0.03384** | down |
| T3 | 4-O-Methylgallic acid | **9.7733** | **0.049782** | up |
| T3 | Syringic acid | **5.382** | **0.002271** | up |
| T3 | Thromboxane B2 | **2.0812** | **0.025648** | up |
| T3 | Deoxycholic acid | **16.55** | 0.39812 | up |
| T3 | N-Acetylarylamine | **6.8251** | 0.37556 | up |
| T3 | Cyclohexylamine | **5.9746** | 0.36514 | up |
| T3 | Chenodeoxycholic acid | **5.017** | 0.46301 | up |
| T3 | Cholic acid | **4.6521** | 0.45266 | up |
| T3 | Taurochenodesoxycholic acid | **4.6056** | 0.38992 | up |
| T3 | m-Coumaric acid | **3.2378** | 0.25328 | up |
| T3 | LysoPC(16:0) | **0.32497** | 0.15085 | down |
| T3 | LysoPE(16:0/0:0) | **0.38351** | 0.15082 | down |
| T3 | Pantothenol | **0.39038** | 0.41059 | down |
| T3 | 2-Hydroxycaproic acid | **2.2387** | 0.14026 | up |
| T3 | Leucinic acid | **2.0881** | 0.11492 | up |
| T3 | Dodecanedioic acid | 1.9924 | **0.002599** | up |
| T3 | L-Histidine | 1.2536 | **0.003478** | up |
| T3 | Caproic acid | 1.5282 | **0.005499** | up |
| T3 | D-Glutamine | 1.7829 | **0.009623** | up |
| T3 | L-Lysine | 1.253 | **0.010283** | up |
| T3 | Caprylic acid | 1.7849 | **0.010429** | up |
| T3 | Citrulline | 1.3535 | **0.011151** | up |
| T3 | N-Acetylhistidine | 1.1863 | **0.018762** | up |
| T3 | Arachidonic acid | 0.74768 | **0.028951** | down |
| T3 | Carnosine | 1.1386 | **0.030143** | down |
| T3 | gamma-Glutamylleucine | 1.174 | **0.037647** | up |
| T3 | Pyroglutamic acid | 1.1365 | **0.040707** | up |

*^1^ T1, day 7 before transportation; T2, day 8 after transportation; T3, day 14 after transportation;*

*^2^ Bold numbers indicate fold change (FC) > 2 or FC < 0.5;*

*^3^ Bold numbers indicate P-value < 0.05.*

**TABLE S2 |** List of loading values for microbiome and metabolomics correlation at T2 and T3^1^.

| **T2: TS vs. TS+TA** | | | | | |
| --- | --- | --- | --- | --- | --- |
| X | Loading_1 | Loading_2 | Y | Loading_1 | Loading_2 |
| Prevotella | 0.340872 | -0.13702 | L-Valine | -0.02404 | 0.2142 |
| Tyzzerella | 0.356186 | 0.032664 | Metronidazole | 0.11371 | -0.18169 |
| Allobaculum | -0.26884 | -0.23421 | Miconazole | 0.078489 | -0.1792 |
| Alloprevotella | 0.344529 | 0.067785 | Indoxyl sulfate | 0.169357 | -0.08919 |
| Halomonas | 0.343373 | 0.069651 | Indoleacetic acid | 0.090295 | 0.16188 |
| Erysipelotrichaceae_UCG-003 | -0.01671 | 0.289182 | Oleamide | 0.01058 | 0.182068 |
| Cetobacterium | 0.120448 | -0.25754 | 3-Hydroxyphenylacetic acid | 0.108869 | -0.14583 |
| Fusobacterium | 0.183943 | -0.20752 | trans-Aconitic acid | 0.103791 | -0.1431 |
| Faecalibaculum | -0.19233 | -0.19789 | Phenylacetic acid | 0.064088 | -0.16258 |
| Dubosiella | 0.109333 | -0.24449 | Leucinic acid | 0.014099 | 0.172234 |
| Candidatus_Arthromitus | -0.01898 | 0.266683 | Indole-3-methyl acetate | 0.064853 | 0.157931 |
| Peptostreptococcus | -0.10407 | -0.24412 | Glyceraldehyde | 0.116484 | 0.122561 |
| [Eubacterium]_brachy_group | 0.12384 | -0.22848 | Hippuric acid | 0.072022 | -0.14383 |
| Slackia | 0.245891 | 0.013146 | 9,10-DHOME | 0.156257 | -0.03427 |
| Escherichia-Shigella | -0.00069 | 0.242432 | Alpha-Hydroxyisobutyric acid | 0.156277 | -0.03406 |
| Collinsella | 0.207138 | 0.10092 | Uric acid | 0.129442 | 0.088984 |
| Sellimonas | 0.145537 | -0.17292 | 5-KETE | 0.155894 | 0.00751 |
| Holdemanella | -0.08133 | 0.20821 | Palmitoleic acid | 0.15522 | -0.00673 |
| Peptococcus | 0.05979 | -0.20206 | Methylsuccinic acid | 0.135427 | -0.07102 |
| Coriobacteriaceae_UCG-002 | -0.178 | -0.10094 | D-2-Hydroxyglutaric acid | 0.115997 | 0.097637 |
| Fusicatenibacter | -0.14018 | 0.127845 | 16-Hydroxy hexadecanoic acid | 0.151023 | -0.00752 |
| Anaerostipes | -0.05566 | 0.166838 | 4-O-Methylgallic acid | 0.041657 | -0.14422 |
| Romboutsia | 0.16254 | -0.04844 | Pyruvic acid | 0.149128 | -2.12E-05 |
| Solobacterium | -0.05737 | 0.15749 | Trimethylamine N-oxide | 0.102328 | -0.10827 |
| Enterococcus | 0.15462 | -0.05808 | Dodecanoic acid | 0.144866 | 0.027481 |
| Turicibacter | 0.145065 | 0.076089 | Benzoic acid | 0.137389 | 0.052831 |
| Streptococcus | 0.095716 | 0.132319 | 3-Indolebutyric acid | -0.0941 | -0.11252 |
| Peptoclostridium | 0.077207 | 0.137876 | N-Acetylornithine | 0.133977 | -0.05934 |
| Adlercreutzia | -0.08689 | -0.13147 | Dibutyl phthalate | 0.130992 | 0.065646 |
| Weissella | -0.09381 | -0.11769 | Alpha-Linolenic acid | 0.14591 | 0.011937 |
| Catenibacterium | 0.004512 | 0.137946 | Glycyl-Isoleucine | 0.137884 | -0.04675 |
| Anaerobiospirillum | 0.056482 | 0.111502 | N-Acetylarylamine | 0.139763 | 0.039894 |
| [Ruminococcus]_gnavus_group | 0.005767 | 0.120412 | 20-Hydroxyeicosatetraenoic acid | 0.116915 | 0.085951 |
| [Ruminococcus]_gauvreauii_group | 0.029514 | -0.11135 | Linoleoyl ethanolamide | 0.112603 | 0.090751 |
| Lactobacillus | 0.045771 | -0.10368 | Phthalic acid | 0.135197 | 0.051286 |
| Clostridium_sensu_stricto_1 | -0.09458 | -0.05709 | L-Lactic acid | 0.122639 | 0.076129 |
| Bifidobacterium | -0.00701 | -0.1096 | Arachidonic acid | 0.127753 | 0.065856 |
| Blautia | -0.05137 | 0.091614 | L-Glutamic acid | -0.00986 | -0.14311 |
| [Ruminococcus]_torques_group | -0.06726 | -0.07216 | 3-Cresotinic acid | 0.143377 | 0.002153 |
| Parasutterella | 0.045132 | -0.03514 | Syringic acid | 0.057095 | -0.12945 |
| Faecalibacterium | 0.041199 | -0.00675 | L-Glutamine | 0.035836 | -0.13643 |
| Subdoligranulum | 0.039351 | -0.00876 | Caproic acid | 0.123151 | 0.067221 |
| Aeromonas | 0.031757 | 0.010046 | Oleic acid | 0.139773 | -0.01135 |
|  |  |  | Caprylic acid | 0.137822 | 0.02079 |
|  |  |  | Biotin | -0.06649 | -0.12148 |
|  |  |  | Alpha-N-Phenylacetyl-L-glutamine | 0.074296 | -0.11657 |
|  |  |  | gamma-Asarone | 0.125992 | 0.054517 |
|  |  |  | Creatine | 0.069255 | -0.11609 |
|  |  |  | Lenticin | 0.112638 | 0.07427 |
|  |  |  | Taurochenodesoxycholic acid | 0.029696 | 0.131363 |
|  |  |  | Capric acid | 0.132052 | -0.01117 |
|  |  |  | Hypoxanthine | -0.00933 | 0.130366 |
|  |  |  | D-Glucurono-6,3-lactone | 0.076495 | -0.10576 |
|  |  |  | 2-Hydroxycinnamic acid | 0.077136 | -0.10399 |
|  |  |  | Phytosphingosine | 0.091822 | 0.089175 |
|  |  |  | Dihydrothymine | 0.090762 | -0.08884 |
|  |  |  | 6-Methylquinoline | 0.033639 | 0.118482 |
|  |  |  | 13-L-Hydroperoxylinoleic acid | 0.107533 | -0.0593 |
|  |  |  | Pantothenic acid | 0.045032 | 0.112949 |
|  |  |  | 4-Coumaryl alcohol | 0.118056 | 0.022622 |
|  |  |  | 3-Hydroxybutyric acid | 0.076281 | -0.09018 |
|  |  |  | m-Coumaric acid | 0.018953 | -0.1162 |
|  |  |  | Prostaglandin B1 | 0.110925 | -0.03911 |
|  |  |  | 2-Aminobenzoic acid | 0.053075 | -0.1045 |
|  |  |  | L-Tryptophan | 0.011737 | 0.116284 |
|  |  |  | p-Hydroxymandelic acid | 0.063641 | 0.097544 |
|  |  |  | Indoleacrylic acid | 0.011505 | 0.115877 |
|  |  |  | trans-Cinnamic acid | 0.066687 | 0.09511 |
|  |  |  | 9-HODE | 0.108066 | -0.04213 |
|  |  |  | L-Phenylalanine | 0.088017 | 0.074377 |
|  |  |  | Inosine | 0.024336 | -0.11255 |
|  |  |  | 5-Hydroxyindoleacetic acid | 0.065661 | 0.093298 |
|  |  |  | D-Glutamine | -0.03843 | -0.10714 |
|  |  |  | 2-Hydroxycaproic acid | -0.02719 | 0.109712 |
|  |  |  | Niacinamide | 0.109494 | -0.02749 |
|  |  |  | Acetaminophen | 0.072686 | -0.08536 |
|  |  |  | Terephthalic acid | 0.060184 | 0.094285 |
|  |  |  | Prostaglandin F2a | 0.107649 | 0.02579 |
|  |  |  | Phosphorylcholine | 0.076023 | 0.07909 |
|  |  |  | Deoxycholic acid | 0.10673 | -0.01281 |
|  |  |  | Indole-3-carboxaldehyde | 0.030427 | 0.102781 |
|  |  |  | Isoquinoline | -0.07805 | -0.07333 |
|  |  |  | Indole | 0.024232 | 0.104045 |
|  |  |  | 2-Hydroxy-2-methylbutyric acid | 0.079777 | -0.0709 |
|  |  |  | Tetradecanedioic acid | 0.000552 | -0.10269 |
|  |  |  | 2,3-Dihydroxytoluene | -0.03138 | -0.09754 |
|  |  |  | N-Acetyl-L-alanine | 0.091067 | 0.041192 |
|  |  |  | Octadecanedioic acid | 0.07178 | -0.06899 |
|  |  |  | Dodecanedioic acid | -0.03466 | -0.09299 |
|  |  |  | N-acetyl-5-aminosalicylic acid | 0.093009 | 0.028423 |
|  |  |  | Hydroxyphenyllactic acid | 0.029334 | 0.092557 |
|  |  |  | 2-Piperidinone | 0.092385 | 0.029299 |
|  |  |  | Salicylic acid | 0.087437 | -0.03598 |
|  |  |  | LysoPC(16:0) | 0.087696 | 0.034122 |
|  |  |  | Phenol | 0.081644 | 0.046186 |
|  |  |  | Linoleic acid | 0.091746 | -0.01598 |
|  |  |  | Cholic acid | 0.090422 | -0.01289 |
|  |  |  | But-2-enoic acid | -0.02892 | 0.08537 |
|  |  |  | L-Carnitine | 0.070681 | -0.05587 |
|  |  |  | 4,6-Dihydroxyquinoline | 0.031361 | 0.084461 |
|  |  |  | Phosphoric acid | -0.0286 | 0.084955 |
|  |  |  | LysoPE(16:0/0:0) | 0.080621 | 0.037553 |
|  |  |  | 2,4-Dihydroxybenzoic acid | 0.083302 | 0.027517 |
|  |  |  | Methacholine | -0.05093 | -0.07089 |
|  |  |  | Palmitic amide | 0.051482 | 0.066362 |
|  |  |  | Homovanillic acid | 0.040892 | -0.07276 |
|  |  |  | 4-Nitrophenol | 0.009551 | -0.07977 |
|  |  |  | gamma-Glutamylleucine | 0.02905 | -0.07215 |
|  |  |  | Hexadecanedioic acid | 0.053302 | -0.05026 |
|  |  |  | Glutaconic acid | -0.02134 | -0.06992 |
|  |  |  | Carnosine | 0.066064 | -0.02909 |
|  |  |  | Adipic acid | 0.049527 | 0.05014 |
|  |  |  | Citric acid | -0.04931 | 0.047191 |
|  |  |  | L-Isoleucine | 0.06266 | 0.024107 |
|  |  |  | 4-Hydroxybenzaldehyde | 0.038768 | -0.05321 |
|  |  |  | Citrulline | -0.06485 | 0.006277 |
|  |  |  | Ureidopropionic acid | 0.064823 | 0.002953 |
|  |  |  | N-Propionylmethionine | 0.030365 | 0.056455 |
|  |  |  | N-Acetylhistidine | 0.063872 | -0.00492 |
|  |  |  | Melatonin | 0.022993 | -0.05601 |
|  |  |  | Kynurenic acid | -0.03429 | 0.045557 |
|  |  |  | Indolelactic acid | 0.044641 | -0.03515 |
|  |  |  | Piperidine | 0.051806 | 0.022611 |
|  |  |  | 3-Hydroxyvaleric acid | 0.041325 | -0.03672 |
|  |  |  | Phenylacetylglycine | 0.042297 | -0.02597 |
|  |  |  | 4-Hydroxyproline | -0.04022 | 0.025381 |
|  |  |  | L-Arginine | 0.030522 | -0.03252 |
|  |  |  | Cyclohexylamine | 0.039515 | -0.01567 |
|  |  |  | 16(R)-HETE | 0.00275 | -0.04203 |
|  |  |  | L-Proline | 0.027952 | -0.03013 |
|  |  |  | Spermidine | 0.02905 | 0.028685 |
|  |  |  | (+/-)-2-Hydroxy-4-(methylthio)butanoic acid | -0.01276 | 0.038687 |
|  |  |  | Glutaric acid | 0.017738 | -0.03665 |
|  |  |  | Pipecolic acid | 0.028998 | 0.028138 |
|  |  |  | 4-Methoxyphenylacetic acid | -0.01478 | 0.032855 |
|  |  |  | L-Lysine | -0.01939 | 0.027468 |
|  |  |  | Taurine | 0.009838 | 0.029957 |
|  |  |  | Thromboxane B2 | 0.016345 | -0.02676 |
|  |  |  | Phenyllactic acid | -0.00997 | 0.026664 |
|  |  |  | Indole-3-propionic acid | 0.024615 | -0.01401 |
|  |  |  | Pyroglutamic acid | 0.00466 | -0.02715 |
|  |  |  | Pantothenol | 0.022016 | -0.01277 |
|  |  |  | L-Histidine | -0.02144 | -0.00904 |
|  |  |  | Chenodeoxycholic acid | -0.00876 | -0.02091 |
|  |  |  | (±)-2-(1-Methylpropyl)-4,6-dinitrophenol | -0.01703 | -0.01436 |
|  |  |  | LysoPE(18:2(9Z,12Z)/0:0) | 0.01428 | 0.009664 |
|  |  |  | 11,12-Epoxyeicosatrienoic acid | -0.01078 | 0.004631 |
| **T3: TS vs. TS+TA** | | | | | |
| X | Loading_1 | Loading_2 | Y | Loading_1 | Loading_2 |
| Faecalibacterium | -0.18487 | -0.23457 | Phenol | -0.1424 | -0.19026 |
| [Ruminococcus]_gauvreauii_group | -0.28683 | 0.048416 | 6-Methylquinoline | -0.20488 | -0.08218 |
| Slackia | -0.15542 | -0.24587 | Piperidine | -0.12888 | 0.178632 |
| Prevotella | -0.15302 | -0.24571 | L-Isoleucine | -0.13599 | 0.17215 |
| Fusobacterium | -0.1063 | -0.26054 | Indolelactic acid | -0.00761 | -0.21261 |
| Sellimonas | -0.1696 | 0.223495 | Indole | -0.2041 | -0.05215 |
| Alloprevotella | -0.12701 | -0.2478 | N-Propionylmethionine | -0.08307 | 0.190107 |
| Parasutterella | -0.12624 | -0.24786 | Indoleacrylic acid | -0.19652 | -0.04932 |
| [Eubacterium]_brachy_group | -0.16151 | -0.22599 | L-Tryptophan | -0.19605 | -0.04969 |
| Blautia | -0.24667 | 0.122963 | Spermidine | 0.194039 | 0.024028 |
| Catenibacterium | -0.19192 | -0.19686 | trans-Cinnamic acid | -0.13864 | 0.129724 |
| Holdemanella | -0.24297 | -0.12766 | 3-Hydroxybutyric acid | -0.00929 | 0.188866 |
| [Ruminococcus]_gnavus_group | -0.14924 | -0.2285 | L-Glutamine | -0.02068 | 0.186991 |
| Fusicatenibacter | -0.16279 | 0.21384 | Thromboxane B2 | 0.125406 | 0.128875 |
| Bifidobacterium | 0.205758 | -0.17026 | Indole-3-carboxaldehyde | -0.17616 | -0.03469 |
| [Ruminococcus]_torques_group | -0.22196 | 0.103918 | L-Valine | -0.01589 | -0.17371 |
| Lachnoclostridium | -0.23089 | 0.066553 | Lenticin | -0.11026 | -0.12574 |
| Brevundimonas | -0.09204 | 0.20922 | gamma-Asarone | -0.00504 | -0.16542 |
| Faecalibaculum | 0.188057 | -0.12785 | 2,3-Dihydroxytoluene | -0.15103 | -0.05446 |
| Dorea | -0.22665 | -0.01047 | L-Phenylalanine | -0.14842 | 0.052736 |
| Allobaculum | 0.176606 | 0.12095 | 2,4-Dihydroxybenzoic acid | -0.15416 | -0.03071 |
| Collinsella | -0.20097 | -0.07189 | Tetradecanedioic acid | 0.154158 | 0.022313 |
| Peptoclostridium | -0.20651 | 0.051068 | D-Glutamine | 0.065543 | 0.140333 |
| Subdoligranulum | -0.17747 | 0.099647 | 2-Hydroxycaproic acid | 0.140599 | -0.05476 |
| Stenotrophomonas | -0.09111 | 0.180711 | Prostaglandin F2a | 0.058266 | 0.138605 |
| Dubosiella | 0.183774 | -0.07991 | 5-Hydroxyindoleacetic acid | -0.14274 | -0.03568 |
| Sphingobium | -0.09653 | 0.174787 | 2-Piperidinone | 0.080233 | -0.12299 |
| Acinetobacter | -0.09231 | 0.16549 | L-Histidine | 0.098663 | 0.104214 |
| Clostridium_sensu_stricto_1 | -0.08705 | 0.162637 | 4-Hydroxybenzaldehyde | -0.09314 | -0.10811 |
| Adlercreutzia | -0.11567 | 0.143267 | 4,6-Dihydroxyquinoline | -0.05378 | -0.13154 |
| Peptostreptococcus | 0.15121 | -0.09044 | Kynurenic acid | -0.07166 | 0.122551 |
| Turicibacter | -0.0945 | 0.108999 | (+/-)-2-Hydroxy-4-(methylthio)butanoic acid | 0.082193 | -0.1137 |
| Anaerostipes | -0.07928 | 0.119619 | But-2-enoic acid | 0.00912 | 0.139596 |
| Cetobacterium | 0.022154 | 0.139397 | 2-Aminobenzoic acid | 0.041826 | 0.132777 |
| Candidatus_Arthromitus | -0.08412 | -0.06346 | Hippuric acid | 0.134012 | -0.03658 |
| Solobacterium | -0.10025 | 0.028745 | Alpha-Hydroxyisobutyric acid | -0.01992 | 0.136761 |
| Coriobacteriaceae_UCG-002 | 0.072555 | 0.051696 | D-2-Hydroxyglutaric acid | 0.041875 | -0.13115 |
| Romboutsia | -0.03607 | 0.080076 | Homovanillic acid | -0.09293 | -0.09988 |
| Tyzzerella | -0.03695 | 0.063225 | 4-Methoxyphenylacetic acid | -0.01958 | -0.13462 |
| Escherichia-Shigella | 0.010467 | -0.0656 | D-Glucurono-6,3-lactone | -0.0308 | 0.130826 |
| Streptococcus | -0.03601 | 0.04737 | Methylsuccinic acid | 0.014813 | -0.1331 |
| Lactobacillus | -0.00384 | -0.04746 | Biotin | 0.126219 | -0.03814 |
| Halomonas | -0.0136 | -0.02098 | (±)-2-(1-Methylpropyl)-4,6-dinitrophenol | 0.002753 | 0.131341 |
| Enterococcus | -0.01815 | -0.00524 | Prostaglandin B1 | 0.077017 | 0.103154 |
| Dietzia | -0.00272 | 0.014864 | Inosine | 0.08172 | -0.09926 |
|  |  |  | Salicylic acid | 0.117642 | -0.05111 |
|  |  |  | 2-Hydroxycinnamic acid | 0.085679 | -0.09467 |
|  |  |  | Caprylic acid | 0.119679 | 0.043516 |
|  |  |  | 3-Cresotinic acid | 0.048284 | -0.1167 |
|  |  |  | Dodecanedioic acid | 0.107614 | 0.060089 |
|  |  |  | Glutaconic acid | 0.058151 | -0.10831 |
|  |  |  | Phenylacetic acid | 0.118468 | -0.02847 |
|  |  |  | 3-Hydroxyphenylacetic acid | 0.113253 | -0.04243 |
|  |  |  | Phosphorylcholine | 0.009002 | 0.117214 |
|  |  |  | Phenyllactic acid | 0.095619 | -0.06836 |
|  |  |  | Citric acid | -0.08459 | 0.08103 |
|  |  |  | Taurine | -0.08528 | -0.07843 |
|  |  |  | Hexadecanedioic acid | 0.109861 | -0.03414 |
|  |  |  | 9,10-DHOME | -0.10313 | -0.04846 |
|  |  |  | 13-L-Hydroperoxylinoleic acid | 0.113204 | 0.011971 |
|  |  |  | Adipic acid | 0.112624 | 0.006811 |
|  |  |  | Leucinic acid | 0.110913 | -0.00299 |
|  |  |  | Dihydrothymine | 0.104129 | 0.032045 |
|  |  |  | Pyruvic acid | -0.00595 | -0.1085 |
|  |  |  | Dodecanoic acid | 0.066149 | -0.08174 |
|  |  |  | Deoxycholic acid | 0.103807 | -0.01614 |
|  |  |  | Chenodeoxycholic acid | 0.101619 | -0.02582 |
|  |  |  | Cholic acid | 0.102361 | -0.02025 |
|  |  |  | 4-Hydroxyproline | -0.09289 | 0.045413 |
|  |  |  | Metronidazole | 0.101739 | -0.01519 |
|  |  |  | Hypoxanthine | -0.02718 | -0.09914 |
|  |  |  | L-Arginine | 0.055059 | -0.08576 |
|  |  |  | m-Coumaric acid | 0.101481 | 0.007619 |
|  |  |  | 3-Hydroxyvaleric acid | 0.078212 | -0.06448 |
|  |  |  | Octadecanedioic acid | 0.097468 | 0.025845 |
|  |  |  | Glyceraldehyde | 0.062663 | -0.0788 |
|  |  |  | Pantothenic acid | -0.03184 | -0.09473 |
|  |  |  | Indole-3-methyl acetate | -0.09957 | -0.00791 |
|  |  |  | Hydroxyphenyllactic acid | 0.060763 | -0.07907 |
|  |  |  | Creatine | -0.00973 | 0.098228 |
|  |  |  | Taurochenodesoxycholic acid | 0.096948 | -0.01212 |
|  |  |  | Linoleoyl ethanolamide | 0.07418 | 0.063086 |
|  |  |  | Arachidonic acid | -0.08214 | -0.05045 |
|  |  |  | Syringic acid | 0.075495 | 0.059098 |
|  |  |  | 11,12-Epoxyeicosatrienoic acid | 0.074487 | 0.058909 |
|  |  |  | Terephthalic acid | -0.02377 | 0.090388 |
|  |  |  | Pipecolic acid | -0.09165 | 0.016404 |
|  |  |  | 20-Hydroxyeicosatetraenoic acid | 0.028952 | -0.08812 |
|  |  |  | Acetaminophen | 0.089533 | -0.00237 |
|  |  |  | L-Carnitine | -0.06381 | 0.062546 |
|  |  |  | p-Hydroxymandelic acid | 0.04627 | -0.0749 |
|  |  |  | Miconazole | 0.083908 | 0.019807 |
|  |  |  | Isoquinoline | 0.047128 | -0.06892 |
|  |  |  | Indoxyl sulfate | 0.018084 | -0.08093 |
|  |  |  | 16(R)-HETE | 0.072747 | 0.037375 |
|  |  |  | Melatonin | 0.054514 | -0.06081 |
|  |  |  | Oleic acid | 0.059517 | 0.055518 |
|  |  |  | N-Acetylhistidine | 0.019763 | 0.077131 |
|  |  |  | 3-Indolebutyric acid | 0.04936 | -0.06173 |
|  |  |  | Phytosphingosine | -0.00304 | -0.07891 |
|  |  |  | Glycyl-Isoleucine | 0.067928 | -0.0402 |
|  |  |  | Trimethylamine N-oxide | 0.074709 | -0.02411 |
|  |  |  | Phosphoric acid | 0.016118 | 0.075041 |
|  |  |  | Indoleacetic acid | -0.06405 | -0.0423 |
|  |  |  | L-Glutamic acid | 0.0216 | 0.073243 |
|  |  |  | Phenylacetylglycine | 0.041863 | -0.06357 |
|  |  |  | gamma-Glutamylleucine | -0.01827 | 0.073601 |
|  |  |  | Uric acid | 0.06431 | -0.03999 |
|  |  |  | 4-O-Methylgallic acid | 0.074332 | -0.00732 |
|  |  |  | N-Acetylornithine | -0.02954 | -0.06543 |
|  |  |  | Palmitoleic acid | 0.007701 | -0.06934 |
|  |  |  | L-Lysine | 0.035416 | 0.057164 |
|  |  |  | trans-Aconitic acid | 0.054587 | -0.03563 |
|  |  |  | Indole-3-propionic acid | 0.0162 | 0.062061 |
|  |  |  | L-Lactic acid | -0.02063 | -0.05987 |
|  |  |  | Caproic acid | 0.05569 | 0.028698 |
|  |  |  | Linoleic acid | 0.060292 | -0.00972 |
|  |  |  | Glutaric acid | 0.007868 | -0.0578 |
|  |  |  | N-Acetyl-L-alanine | -0.01949 | -0.05433 |
|  |  |  | Ureidopropionic acid | 0.028934 | -0.04769 |
|  |  |  | Alpha-Linolenic acid | 0.034448 | -0.04188 |
|  |  |  | Alpha-N-Phenylacetyl-L-glutamine | 0.035064 | -0.04054 |
|  |  |  | Niacinamide | -0.05287 | 0.005814 |
|  |  |  | Dibutyl phthalate | -0.01873 | -0.04968 |
|  |  |  | Phthalic acid | -0.01502 | -0.05027 |
|  |  |  | Citrulline | 0.026428 | 0.045042 |
|  |  |  | 4-Coumaryl alcohol | 0.046167 | 0.024336 |
|  |  |  | Benzoic acid | -0.01177 | -0.04977 |
|  |  |  | Pyroglutamic acid | -0.04665 | -0.01906 |
|  |  |  | 4-Nitrophenol | -0.02991 | 0.040439 |
|  |  |  | 2-Hydroxy-2-methylbutyric acid | 0.045198 | -0.02113 |
|  |  |  | Cyclohexylamine | -0.00966 | 0.045442 |
|  |  |  | N-Acetylarylamine | -0.0062 | 0.045019 |
|  |  |  | 16-Hydroxy hexadecanoic acid | 0.011557 | -0.04077 |
|  |  |  | Palmitic amide | 0.012056 | -0.0406 |
|  |  |  | Pantothenol | 0.033019 | 0.024795 |
|  |  |  | L-Proline | -0.03708 | 0.016358 |
|  |  |  | N-acetyl-5-aminosalicylic acid | 0.036538 | 0.011789 |
|  |  |  | Methacholine | -0.0137 | 0.035561 |
|  |  |  | 9-HODE | 0.010656 | 0.03641 |
|  |  |  | Capric acid | 0.008106 | 0.032694 |
|  |  |  | LysoPE(18:2(9Z,12Z)/0:0) | 0.02592 | -0.01618 |
|  |  |  | Carnosine | -0.02372 | 0.01628 |
|  |  |  | 5-KETE | 0.011398 | 0.020796 |
|  |  |  | LysoPE(16:0/0:0) | -0.00602 | -0.01721 |
|  |  |  | Oleamide | 0.014096 | -0.00976 |
|  |  |  | LysoPC(16:0) | -0.00845 | -0.00914 |

*^1^ T2, day 8 after transportation; T3, day 14 after transportation.*
